# The global impact and cost-effectiveness of a melioidosis vaccine

**DOI:** 10.1186/s12916-019-1358-x

**Published:** 2019-07-05

**Authors:** Nantasit Luangasanatip, Stefan Flasche, David A. B. Dance, Direk Limmathurotsakul, Bart J. Currie, Chiranjay Mukhopadhyay, Tim Atkins, Richard Titball, Mark Jit

**Affiliations:** 1 Department of Infectious Disease Epidemiology, Faculty of Epidemiology and Population Health, London School of Hygiene and Tropical Medicine, London, UK; 2 College of Life and Environmental Sciences, University of Exeter, Exeter, UK; 3 Mahidol-Oxford Tropical Medicine Research Unit, Faculty of Tropical Medicine, Mahidol University, Bangkok, Thailand; 4 Lao-Oxford-Mahosot Hospital-Wellcome Trust Research Unit (LOMWRU), Vientiane, Lao People’s Democratic Republic; 5 Centre for Tropical Medicine and Global Health, Nuffield Department of Clinical Medicine, University of Oxford, Oxford, UK; 6 Faculty of Infectious and Tropical Disease, London School of Hygiene and Tropical Medicine, London, UK; 7 Department of Tropical Hygiene, Faculty of Tropical Medicine, Mahidol University, Bangkok, Thailand; 8 Global and Tropical Health Division, Menzies School of Health Research, Darwin, Australia; 9 Department of Infectious Diseases and Northern Territory Medical Program, Royal Darwin Hospital, Darwin, Australia; 10 Department of Microbiology, Kasturba Medical College, Manipal Academy of Higher Education, Manipal, Karnataka India; 11 Defence Science and Technology Laboratory, Salisbury, UK

**Keywords:** Melioidosis, Infections, Vaccine, Cost-effectiveness, Economic evaluation

## Abstract

**Background:**

Every year, 90,000 people may die from melioidosis. Vaccine candidates have not proceeded past animal studies, partly due to uncertainty around the potential market size. This study aims to estimate the potential impact, cost-effectiveness and market size for melioidosis vaccines.

**Methods:**

Age-structured decision tree models with country-specific inputs were used to estimate net costs and health benefits of vaccination, with health measured in quality-adjusted life years (QALYs). Four target groups of people living in endemic regions were considered: (i) people aged over 45 years with chronic renal disease, (ii) people aged over 45 years with diabetes, (iii) people aged over 45 years with diabetes and/or chronic renal disease, (iv) everyone aged over 45 years. Melioidosis risk was estimated using Bayesian evidence synthesis of 12 observational studies. In the base case, vaccines were assumed to have 80% efficacy, to have 5-year mean protective duration and to cost USD10.20–338.20 per vaccine.

**Results:**

Vaccination could be cost-effective (with incremental cost-effectiveness ratio below GDP per capita) in 61/83 countries/territories with local melioidosis transmission. In these 61 countries/territories, vaccination could avert 68,000 lost QALYs, 8300 cases and 4400 deaths per vaccinated age cohort, at an incremental cost of USD59.6 million. Strategy (ii) was optimal in most regions. The vaccine market may be worth USD268 million per year at its threshold cost-effective price in each country/territory.

**Conclusions:**

There is a viable melioidosis vaccine market, with cost-effective vaccine strategies in most countries/territories with local transmission.

**Electronic supplementary material:**

The online version of this article (10.1186/s12916-019-1358-x) contains supplementary material, which is available to authorized users.

## Background

*Burkholderia pseudomallei* is a highly pathogenic gram-negative bacillus that is the causal agent of melioidosis [1]. The bacteria are present mainly in soil and water, and people are infected through inoculation, ingestion and inhalation [2]. A recent study estimated that melioidosis is endemic in 83 countries/territories (hereafter “geographies”), mainly in South-East Asia and sub-Saharan Africa, and that it causes 164,938 cases and 88,979 deaths annually [3]. Major risk factors for infection include diabetes, chronic renal and lung disease, immunosuppression and excess alcohol consumption [4, 5]. The indigenous population in Australia is also at higher risk of melioidosis. *B. pseudomallei* is resistant to many antibiotics, and extended courses of treatment, often using multiple drugs, are typically required [1, 6]. In addition, many cases of melioidosis are advanced when diagnosed and treated, so mortality is high [1].

Consequently, a vaccine could be valuable for the prevention of disease in many parts of the world. Several vaccine candidates have shown promising results in animals but to date no human studies have been conducted [7, 8]. A recent study showed that a melioidosis vaccine could be cost-effective in north-eastern Thailand if it protects for at least 3 years and can reduce both incidence and mortality of melioidosis by at least 80%; the threshold cost-effective price was $1 for vaccinating everyone aged 35 years and $8 for vaccinating diabetics aged over 35 years ([7] and Yoel Lubell, personal communication). However, no economic analyses have been conducted outside this location.

Establishing a cost-effective strategy for the use of a melioidosis vaccine in different countries, and hence the size of the potential global market for the vaccine, is key to unlocking investment into further vaccine development. It can also help guide local decision makers about optimal vaccine use if a vaccine becomes available. Hence, our main objective is to assess the potential impact and cost-effectiveness of different strategies for using a melioidosis vaccine, at a global level.

## Methods

### Overview

We estimated the impact and cost-effectiveness of melioidosis vaccination in 83 geographies where melioidosis is believed to be endemic [3]. In each location, we estimated the burden of melioidosis in three subpopulations at high risk of disease: people aged over 45 years old, diabetics and chronic renal disease patients. To do this, we used a Bayesian model to synthesise information extracted from 12 observational studies reported in a recent systematic review to estimate the relative risk of melioidosis in these groups [3]. These estimated relative risks were applied to our estimates of melioidosis incidence in every geography to quantify the incidence in people with one or more of these risk factors.

Incidence estimates were then combined with geography-specific epidemiological, demographic and clinical parameters to examine the impact of different vaccination strategies. Four target populations for vaccination were examined: (i) people aged over 45 years with chronic renal disease (Vac 1), (ii) people aged over 45 years with diabetes (Vac 2), (iii) people aged over 45 years with diabetes and/or chronic renal disease (Vac 3) and (iv) people aged over 45 years (Vac 4). In each case, vaccination was assumed to be confined to areas identified as having environmental conditions suitable for *B. pseudomallei* in the global burden study [3].

Using these impact estimates, a cost-effectiveness analysis was conducted in each geography by estimating the incremental cost (in USD) per quality-adjusted life year (QALY) gained for each strategy. This analysis considered the cost and QALY loss due to hospitalised and fatal melioidosis, as well as the cost of vaccination.

### Model structure

**Fig. 1:**
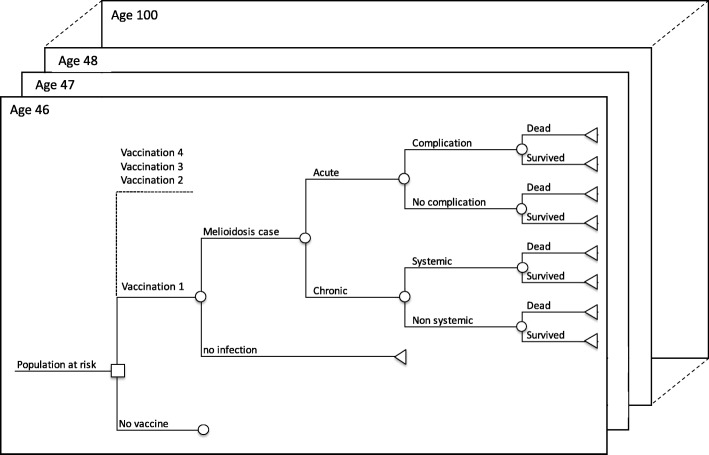
Decision tree showing that vaccination is offered at age 46 and the cohort is followed up until age 100

The disease model assumes that melioidosis is transmitted through contact with the environment (soil, water), since there is no evidence of person-to-person transmission [9]. A geography-specific age-structured static decision tree model with lifetime horizon was developed (Fig. 1) containing four melioidosis endpoints based on clinical acquisition and presentation: (i) acute disease with complications, (ii) acute disease without complications, (iii) chronic disease with systemic illness and (iv) chronic disease without systemic illness [10]. The geography-specific overall mortality due to melioidosis was assumed to be the same as predicted in the global burden study [3].

Patients who survive a melioidosis episode were assumed to return to full health with the same life expectancy as the general population but may experience another episode later in life. We assumed that life expectancy was the same for anyone living in a particular country, due both to lack of data about life expectancy in different risk groups as well as to avoid ethical difficulties around discriminating against risk groups with lower life expectancy in economic evaluations [11].

Definitions of the different clinical presentations of melioidosis were adopted from Ketheesan et al. [12] (Additional file 1: Table S1). The total number of cases and deaths per year matched numbers reported in the recent global burden study [3]. A geography-specific life-time cohort model stratified by age groups was developed. The population aged 46 that received vaccination was treated as a single cohort and followed up until death.

### Data and parameter inputs

#### Population at risk: general and environment suitable area

We assumed that melioidosis cases were confined to people living in a geographical region environmentally suitable for melioidosis transmission. Environmental suitability was defined in the same way as in Limmathurotsakul et al., i.e. any location in the fifth percentile of positive occurrence records (or at least 0.1567 on the 0–1 predicted environmental suitability scale) in that paper [3]. Areas matching the definition (by 5 km × 5 km quadrants) were matched to the population density map derived from Global Rural-Urban Mapping Project (GRUMP) 2010 adjusted for population per geography in the 2015 United Nations World Population Prospects to estimate geography-specific population at risk for melioidosis [3, 13].

#### Relative risk of melioidosis for different risk factors

We estimated the relative risk of contracting melioidosis for three risk factors: age over 45 years, diabetes and chronic renal disease. Studies reporting the incidence of melioidosis identified in a recent systematic review were included in this analysis [3]. Of the 20 observational studies (Additional file 1: Text S1) reporting the number of cases with different potential risk factors, only 11 reported the proportion of cases related to either diabetes, chronic renal disease, or age group [4, 5, 14–22]. All of these studies reported the number of diabetic cases while ten studies revealed the number of cases with chronic renal disease. Six studies reported the number of cases aged over either 40, 45, 50 or 55 years.

We synthesised information in each study to determine global relative risks, assuming that they do not differ across studies and geographies. A Poisson log-likelihood function was used to fit estimates of relative risks to observed counts of melioidosis cases in each of eight risk subgroups (i.e. people with no risk, diabetes, chronic renal disease, age over 45 years or any combination of these) within a Bayesian framework. We assumed that the prevalence of risk factors in each study was the same as that in the general population in the same geography. Markov Chain Monte Carlo (MCMC) was then used to estimate the marginal probabilities for the relative risk of melioidosis in different groups. The risk of melioidosis in people with a combination of risk factors was assumed to be the product of the marginal risks. The estimated relative risks were then compared with the population with no risk factors defined as the population aged under 45 without either diabetes or chronic renal disease. Although there are slight differences in the age threshold for being high risk across the studies (ranging from 40 to 55), we assumed that the threshold across all studies was 45 years. (Additional file 1: Figure S1 (a-c), Table S2 and Text S2).

#### Risk of melioidosis and risk of death

The incidence of melioidosis in environmentally suitable areas in each geography for people with either single or combined risk factors of being over 45 years, diabetic and/or having chronic renal disease was estimated from the predicted relative risks. (Additional file 1: Table S3 for details). The risk of each of the four melioidosis endpoints (acute disease with and without complication; chronic disease with and without systemic illness) was estimated by expert elicitation due to paucity of data in the literature (Additional file 1: Text S3 for the questionnaire used). These parameters were assumed to be the same for every geography. The parameters to be estimated were independently presented to four clinicians with experience of melioidosis (authors D.A.B.D., D.L., B.J.C. and C.M.) to estimate the proportion and risk of death for each of the melioidosis conditions, and the fraction of intensive care admission among acute melioidosis with complication patients. The median, minimum and maximum values from the four respondents were fitted with gamma distribution. Geography-specific overall number of cases and deaths due to melioidosis were obtained from the incidence and mortality predicted each year in the global burden study [3].

#### Demographic data

In most geographies, age-specific life expectancy was estimated from World Health Organisation life tables, 2015 [23]. Three countries without life tables (Sierra Leone, Guinea-Bissau and Gambia) were matched to life tables in Uganda, Somalia and Liberia respectively based on geographical and income similarity. Life tables for Hong Kong were obtained from the city’s National Census and Statistics Department [24]. The geography-specific prevalence of diabetes and chronic renal disease were respectively obtained from the International Diabetes Federation’s Diabetes Atlas, 2012 (available at geography-level) and a systematic review (available at regional level) [25, 26], assuming these figures are homogeneous across geographical regions and age groups, within the same geography.

#### Vaccination strategies and cost-effectiveness analysis

The costs of melioidosis treatment were estimated by micro-costing assuming melioidosis cases were treated with regimens in compliance with treatment guidelines [10] (see Additional file 1: Text S4 for details). Because licenced melioidosis vaccines are not currently available, we assumed the total costs of vaccination to be USD10.2, 43.5, and 338.2 in low-, middle- and high-income countries, respectively. This was based on the total cost of purchasing and delivering a 2-dose course of human papillomavirus (HPV) vaccines in countries from different income levels [27]. Health-related quality of life due to melioidosis infections regardless of endpoint was assumed to be similar to bacteraemia [28]. Total QALY loss was then estimated by multiplying the length of hospitalisation for the melioidosis endpoint with the quality of life weight, adjusting for population norms (i.e. the fact that the baseline population is not in perfect health) [29]. Vaccine efficacy was assumed to be 80% (for an all-or-nothing vaccine), with protection waning exponentially with a mean duration of 5 years in the base case [7].

For each vaccine strategy (Vac 1-Vac 4), we assessed the incremental costs incurred and QALYs gained to calculate the incremental cost-effectiveness ratio (ICER) of each vaccination strategy in every geography. The Commission on Macroeconomics and Health suggests that an intervention is considered to be very cost-effective if its ICER is less than the gross domestic product (GDP) per capita [30]. For each geography, we identified the strategies (Vac 1–Vac 4) that were cost-effective under this definition. We also estimated the maximum price that could be paid for vaccination for the most expensive strategy (vaccinate everyone over 45 in an environmentally suitable area) to be cost-effective. In addition, we conducted a regional analysis by aggregating costs and vaccine effects over five United Nations regions: East Asia and the Pacific (EAP), South Asia (SA), sub-Saharan Africa (SAF), Latin America and the Caribbean (LAC) and the Middle East and North Africa (MENA). We performed probabilistic sensitivity analyses to examine the robustness of the results. A societal perspective was employed using a 3% discount rate for both costs and health benefits. A sensitivity analysis with 50% protective efficacy and 5 years protection was performed.

## Results

**Fig. 2:**
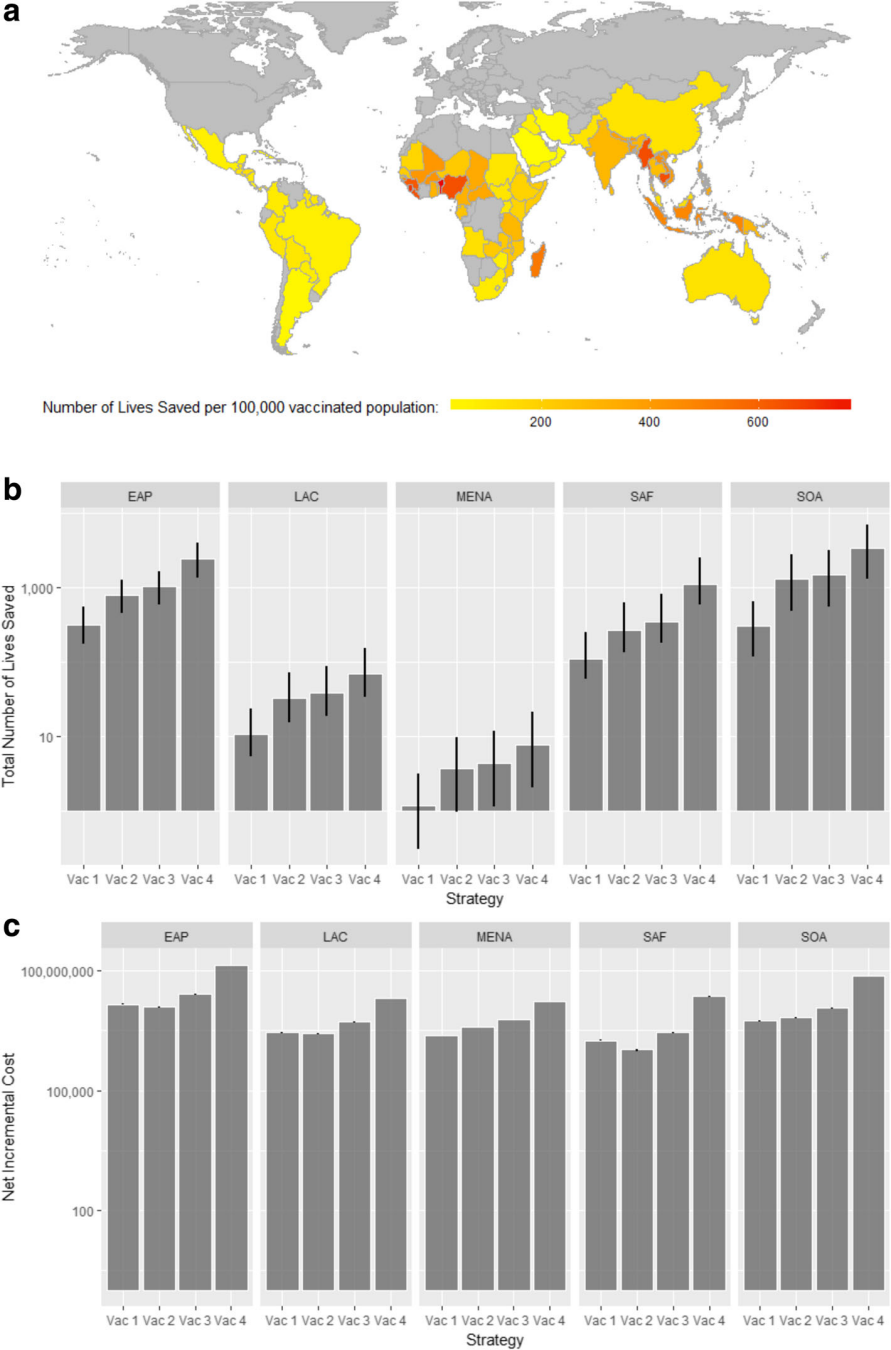
Results of the base case analysis assuming 80% vaccine protective efficacy with 5-year mean protective duration. **a** Map showing number of lives saved by geography per 100,000 diabetics aged > 45 years vaccinated in environmentally suitable regions (strategy Vac 2). **b** Bar plot showing the total number of deaths averted by region for each vaccination strategy. **c** Bar plot showing the net costs of vaccination by region for each vaccination strategy

**Fig. 3:**
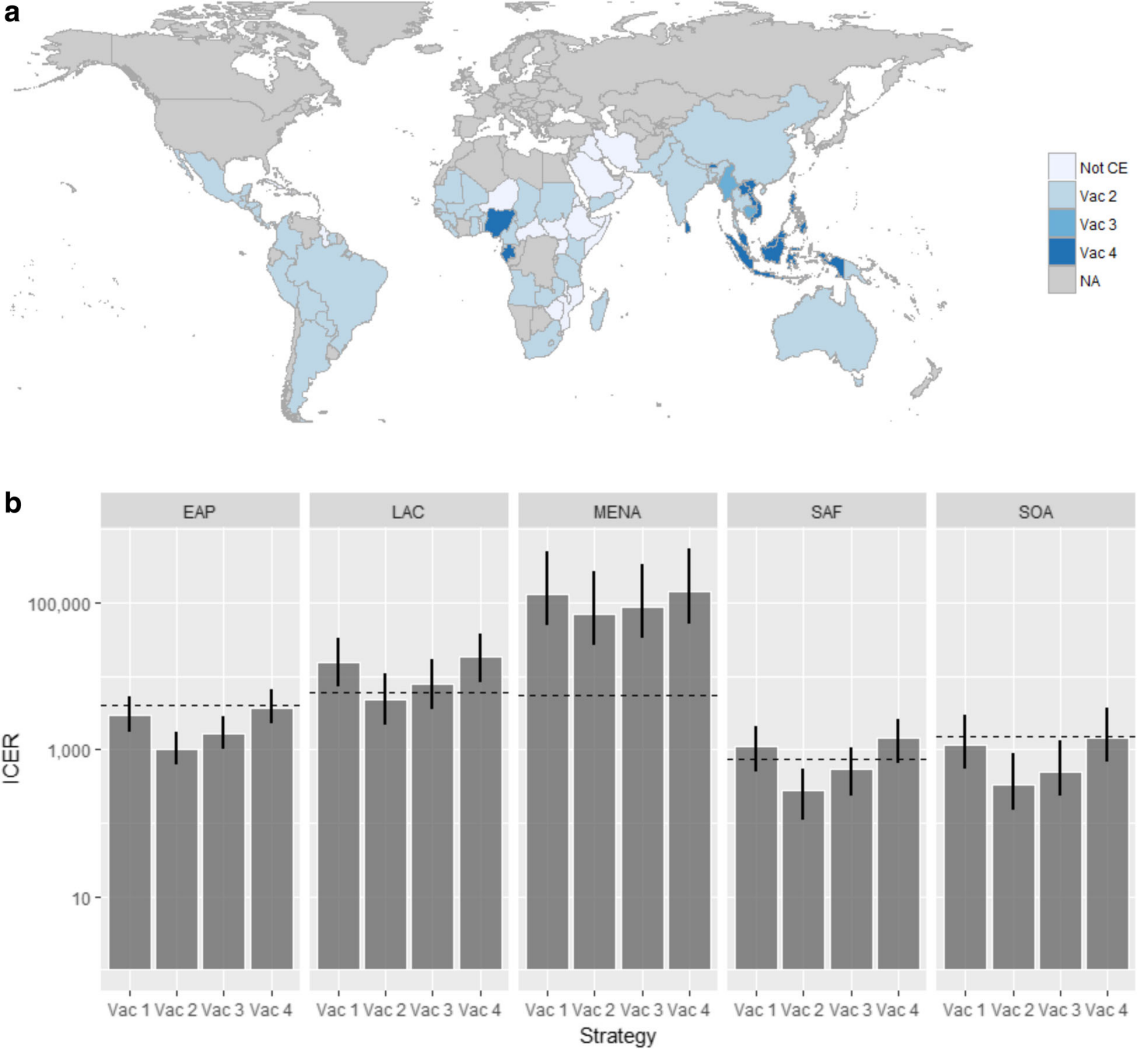
Geography-specific optimal cost-effective vaccination strategy and cost-effectiveness results of base case analysis by region. **a** Optimal cost-effective vaccination strategy by geography. **b** Incremental cost-effectiveness ratio for each vaccine strategy by region. Strategies are vaccinating (i) people over 45 years with chronic renal disease (Vac 1), (ii) people aged over 45 years with diabetes (Vac 2), (iii) people aged over 45 years with diabetes and/or chronic renal disease (Vac 3) and (iv) people aged over 45 years (Vac 4)

**Fig. 4:**
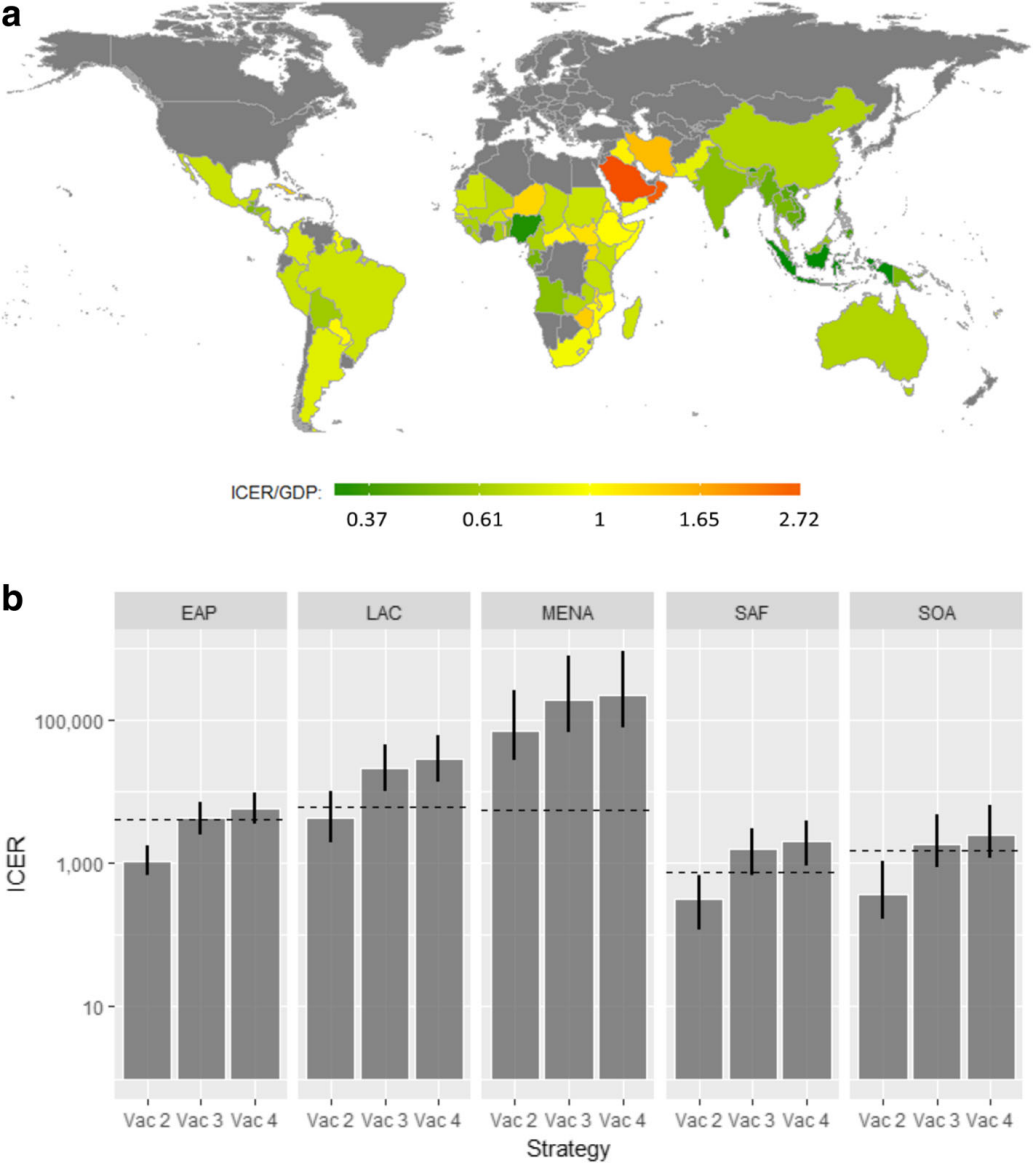
Geography-specific incremental cost-effectiveness ratio (ICER) per GDP per capita and comparative effectiveness results of base case analysis by region. **a** Incremental cost-effectiveness ratio (ICER) of vaccinating the population over 45 years with diabetes (strategy Vac 2), as a proportion of each geography’s GDP per capita. **b** ICER by region of each strategy compared to the next best strategy (Vac 2 compared with no vaccination, Vac 3 compared with Vac 2, and Vac 4 compared Vac 3)

Our synthesis of evidence from 12 observational studies indicates that diabetes is the risk factor for melioidosis with the highest relative risk, followed by age over 45 years and chronic renal disease (Table 1). In the regional analysis, vaccinating diabetics aged over 45 years living in environmentally suitable areas for melioidosis (Vac 2) would be cost-effective in EAP, SAF and SOA (Fig. 2; see Additional file 1: Table S4 and Figure S2 for detailed results). However, those vaccination strategies would not be cost-effective in LAC and MENA. In the country/territory-level analysis, vaccinating diabetics aged over 45 years (Vac 2) is a cost-effective strategy in 45 geographies. Of these, vaccinating individuals aged over 45 years with either diabetes or chronic renal diseases (Vac 3) is the optimum strategy in six geographies, while vaccinating everyone aged over 45 years with or without diabetes/chronic renal disease (Vac 4) is optimal in ten geographies (Additional file 1: Table S5). However, in 22 geographies, none of the strategies tested (Vac 1-Vac 4) are cost-effective (Fig. 3). Figure 4 shows the cost-effectiveness of vaccinating the diabetic population aged over 45 years (Vac 2) in each geography, as a ratio of GDP per capita.

**Table 1:** Parameters used in the analysis

	Values			References
Risk of melioidosis (baseline)	See Additional file 1: Table S3(a)			
Relative risk of melioidosis by risk factor				
- Age > 45 years	2.01 (1.87, 2.15)			Estimated from literature
- Diabetes	6.50 (6.10, 6.93)			Estimated from literature
- Chronic renal disease	1.33 (1.20, 1.46)			Estimated from literature
Probability of each condition given melioidosis infection (3 levels; high-, middle- and low-income geographies)	High	Middle	Low	
Probability of acute disease with complications	0.40	0.50	0.25	Expert opinion
Probability of acute disease without complications	0.48	0.40	0.55	Expert opinion
Probability of chronic disease with systemic illness	0.07	0.05	0.07	Expert opinion
Probability of chronic disease without systemic illness	0.06	0.05	0.12	Expert opinion
Risk of death (relative risk for each condition)				
Baseline risk of death (chronic disease without systemic illness)	Vary between geographies			Estimated
Relative risk for acute disease with complications	16.42			Expert opinion
Relative risk for acute disease without complications	5.33			Expert opinion
Relative risk of chronic disease with systemic illness	5.17			Expert opinion
Probability of intensive care unit admission among acute cases with complication	0.62 (0.48, 0.75)			Expert opinion
Economic parameters				
Resource use				
Length of hospital stay (LOS)	Median (95% CI)			Indian hospital data
Intensive care unit (ICU) for acute disease with complication cases	3.20 (0.58, 10.04)			
General hospitalisation				
- Acute disease with complication	13.85 (1.66, 34.71)			
- Acute disease without complication	14.77 (2.51, 34.35)			
- Chronic disease with systemic illness	18.19 (4.20, 41.61)			
- Chronic disease without systemic illness	15.36 (1.06, 41.81)			
Treatment duration (days)				[10]
- Parenteral regimens	12 (10, 14)			
Meropenem or ceftazidime				
- Oral regimens	112 (84, 140)			
Trimethroprim/sulfamethoxazole or co-amoxiclav				
Costs (USD, 2016)				
Cost of vaccine (complete course) by country income level	Low 10.2 Middle 43.5 High 338.2			[27]
Cost per bed day	WHO-CHOICE			[31]
Cost per ICU bed day				
Ratio between ICU and general bed day by income level	High 2.56 Middle 7.92 Low 13.28			See Additional file 1: Table S3(b)
Antibiotics (per day), children aged < 15 years	High	Middle	Low	
Meropenem	46.78	59.47	N/A	[32, 33]
Ceftazidime	27.89	3.44	3.44	
Trimethoprim/sulfamethoxazole	0.91	0.05	0.05	
Co-amoxiclav	0.71	1.41	1.41	
Antibiotics (per day), adults aged > 15 years	High	Middle	Low	
Meropenem	62.37	79.30	N/A	[32, 33]
Ceftazidime	43.39	5.35	5.35	
Trimethoprim/sulfamethoxazole	1.12	0.06	0.06	
Co-amoxiclav	1.06	2.11	2.11	
Health-related quality of life (utility)				
Bacteraemia	0.36 (0.33, 0.38)			[28]
General population	0.84			[29]
Life expectancy	WHO Life Table			[23]

Of 5.47 billion people across the 83 evaluated geographies, 1.55 billion are predicted to live in environmentally suitable areas for melioidosis transmission [3]. Of these, 457 million are > 45 years old. If the optimal cost-effective strategy in Additional file 1: Table S5 was applied in each of the 61 geographies where one of the vaccine strategies was found to be cost-effective, 5.26 million people would be vaccinated per year, averting on average 69,000 lost QALYs, 8400 cases and 4500 deaths (i.e. 5.09% reduction in total burden) per vaccinated age cohort. Vaccination would cost USD62.1 million per year but save USD2.47 million in treatment costs. The regions in order of the magnitude of projected reductions in cases are EAP, SOA, SAF, LAC and MENA (with reductions in total burden of 6.72%, 5.30%, 3.87%, 3.58% and 1.99% respectively).

Alternatively, if everyone aged 46 living in an environmentally suitability area was vaccinated (Vac 4), then 15.18 million people would be vaccinated per year. This could potentially prevent 13,628 cases of melioidosis and 7386 deaths (9.4% reduction) with a gain of 113,407 QALYs per vaccinated age cohort. If the cost of vaccination was not fixed, then under this scenario the threshold cost of vaccination (averaged across all countries) would be USD18.54, giving a potential market size of USD281.32 million per year (ignoring vaccine distribution costs).

A sensitivity analysis showed that with only 50% vaccine protection, vaccinating diabetics aged over 45 years (Vac 2) is a cost-effective strategy in 43 geographies. In addition, vaccinating individuals aged over 45 years with diabetes and/or chronic renal disease (Vac 3) is the optimum strategy in three geographies, while vaccinating everyone aged over 45 years with or without diabetes/chronic renal disease (Vac 4) is optimal in five geographies. In 32 geopraphies, none of the strategies tested (Vac 1-Vac 4) are cost-effective (Additional file 1: Figure S3(a-g) and Table S6). If the optimal cost-effective strategy was applied in each of the 51 geographies, 3.55 million people would be vaccinated per year, averting on average 35,370 lost QALYs, 4311 cases and 2332 deaths (2.61% reduction) per vaccinated age cohort.

## Discussion

A recent disease burden study suggested that there are around 165,000 cases and 89,000 deaths due to melioidosis every year and that the disease is endemic in at least some parts of 83 geographies. Our impact and cost-effectiveness modelling indicates that a melioidosis vaccine targeted at high-risk populations living in environmentally suitable areas for melioidosis transmission could reduce the burden of this disease in these populations and be cost-effective. To our knowledge, this is the first study evaluating the impact and economic consequences of melioidosis vaccination from a global perspective. In addition, this is the first study systematically quantifying the relative risk of melioidosis in different groups by pooling evidence from a review of observational studies.

Only one cost-effectiveness study of melioidosis vaccination has been published; this was restricted to north-eastern Thailand but also found vaccination to be potentially cost-effective [7]. Our analysis extends this by considering all geographies with endemic melioidosis, a wide range of risk factors based on a synthesis of studies in the literature, and a range of vaccine strategies. By targeting the vaccine to the population at greatest risk, we can ensure that vaccination is cost-effective even when its duration of protection is short (5 years).

In addition, with the trend of increasing prevalence of chronic renal disease and diabetes in some of the endemic countries, the size of the high-risk population could increase considerably in the near future, making the vaccine more cost-effective [25, 26]. The long-term prognosis for these chronic diseases is improving in many countries, again making the protection from a melioidosis vaccine potentially more valuable in the future. On the other hand, melioidosis patients may have better survival prospects due to the better quality and accessibility of treatment [34].

The potential target areas for vaccination were determined from a study that determined environmentally suitable areas for melioidosis with very high spatial resolution (5 × 5 km^2^). However, the environmentally suitable areas generally do not correspond to administrative boundaries between or within countries, especially in large geographies such as Australia, China and India. In practice, it may be easier to target vaccination strategies by administrative units such as provinces or states based on average risks within those units. Improving surveillance systems at both the local and global levels would also strengthen the robustness of data informing such decisions.

This study has several limitations. Firstly, we considered three risk factors for melioidosis in our evaluation: age over 45 years, diabetes and chronic renal disease. These have been consistently identified as some of the most important risk factors for melioidosis in studies. However, other attributes have also been identified as potential risk factors, although they are less consistently reported and/or have smaller reported relative risks. They include male gender, chronic lung disease, thalassaemia, excessive alcohol consumption and being an indigenous Australian [2, 12]. Further data about melioidosis relative risks and prevalence of these risk factors at the country level may help more precisely targeted vaccination strategies that could increase vaccine impact and cost-effectiveness further.

Secondly, many parameters around treatment protocols and costs were estimated for broad categories of geographies stratified by income level. Furthermore, some parameters had to be established through expert elicitation due to limitations in available data. Economic studies at the country/territory level could help establish more reliable estimates of the economic burden of melioidosis.

Moreover, in the absence of explicit cost-effectiveness threshold in most of the evaluated countries, we adopted one GDP per capita to be the threshold which has been widely used in low- and middle-income settings [30]. However, recent modelling evidence suggests that GDP per capita thresholds are much higher than the actual heath opportunity costs in several countries [35, 36]. Hence, our findings may require careful interpretation and ideally supplemented by country-level analyses using local thresholds and deliberative processes.

Lastly, as no melioidosis vaccines have entered human trials to date, we made broad assumptions about the potential cost, protective efficacy and duration of protection afforded by a vaccine. Our assumptions have been fairly conservative: we assumed short duration (5 years), imperfect efficacy (50–80%) and vaccine cost assumptions based on one of the most commercially successful vaccines to date (human papillomavirus vaccine). By doing so, our analysis establishes the minimal characteristics of a vaccine that can be successful in commercial and public health terms for vaccine developers to aim for. Even with 50% protective efficacy assumption in sensitivity analysis, a cost-effective vaccine strategy still exists in 51 out of 83 melioidosis endemic countries. If a melioidosis vaccine is brought to market with longer duration, better efficacy or a lower price, then it will be even more cost-effective than we report.

## Conclusions

In conclusion, our findings establish cost-effective strategies for use of melioidosis vaccines in the majority of environmentally suitable areas for transmission. These results support the case for investors and manufacturers to bring a candidate vaccine to the market, as well as to donors and local decision makers to use such a vaccine at the population level when it becomes available. Our results also help in determining the market size and suitable price points for a vaccine if it becomes available.

## Additional file

Additional file 1: **Table S1.** Definition of clinical terms used in the main text. **Text S1**. Literature used in the studies. **Figure S1.** Detailed results for the Bayesian synthesis of 12 observational studies. **Table S2.** Extracted raw data of number of melioidosis case by subgroup of risk factors from observational studies. **Text S2**. Equation to estimate risk of melioidosis and relative risk associated with each risk factor. **Text S3**. Questionnaire used for elicitation of expert opinion. **Text S4**. Melioidosis treatment and costing details (Additional file 2). **Table S3.** Country-specific parameter inputs. **Table S4.** Incremental cost-effectiveness ratio (ICER) for different vaccination strategies (compared to the next most expensive strategy) by region. **Figure S2.** Incremental cost-effectiveness ratio (ICER) for different vaccination strategies (compared to no vaccination) by region. **Table S5.** Results of the base case analysis by country/territory. **Figure S3.** Results from a sensitivity analysis assuming 50% vaccine protective efficacy. **Table S6.** Results of all countries/territories from the sensitivity analysis, 50% vaccine protective efficacy. (DOCX 895 kb)

Additional file 2: Copy of Data summary LOSR2. (XLSX 9 kb)

## Data Availability

Any dataset produced, used and/or analysed over the course of the current study that is not already in the Additional file is available from the corresponding author on reasonable request.

## Abbreviations

EAP: East Asia and the Pacific
GDP: Gross domestic product
GRUMP: Global Rural-Urban Mapping Project
ICER: Incremental cost-effectiveness ratio
LAC: Latin America and the Caribbean
MCMC: Markov Chain Monte Carlo
MENA: Middle East and North Africa
QALYs: Quality-adjusted life years
SA: South Asia
SAF: Sub-Saharan Africa
