# Supplementary material for: The global impact and cost-effectiveness of a melioidosis vaccine
Source: BMC Med. 2019 Jul 5;17:129. doi: 10.1186/s12916-019-1358-x (PMC6610909; doi:10.1186/s12916-019-1358-x)
Supplement: Supplementary file 1 — Table S1. Definition of clinical terms used in the main text. Text S1. Literature used in the studies. Figure S1. Detailed results for the Bayesian synthesis of 12 observational studies. Table S2. Extracted raw data of number of melioidosis case by subgroup of risk factors from observational studies. Text S2. Equation to estimate risk of melioidosis and relative risk associated with each risk factor. Text S3. Questionnaire used for elicitation of expert opinion. Text S4. Melioidosis treatment and costing details (Additional file 2). Table S3. Country-specific parameter inputs. Table S4. Incremental cost-effectiveness ratio (ICER) for different vaccination strategies (compared to the next most expensive strategy) by region. Figure S2. Incremental cost-effectiveness ratio (ICER) for different vaccination strategies (compared to no vaccination) by region. Table S5. Results of the base case analysis by country/territory. Figure S3. Results from a sensitivity analysis assuming 50% vaccine protective efficacy. Table S6. Results of all countries/territories from the sensitivity analysis, 50% vaccine protective efficacy. (DOCX 895 kb) [file 12916_2019_1358_MOESM1_ESM.docx]

**Additional file 1**

**Title: “*The global impact and cost-effectiveness of a melioidosis vaccine”***

**Nantasit Luangasanatip, Stefan Flasche, David A. B. Dance, Direk Limmathurotsakul, Bart J. Currie, Chiranjay Mukhopadhyay, Tim Atkins, Richard Titball, Mark Jit**

Content list

Table S1. Definition of clinical terms used in the main text

Text S1. Literature used in the studies

Figure S1. Detailed results for the Bayesian synthesis of 12 observational studies

Table S2. Extracted raw data of number of melioidosis case by subgroup of risk factors from observational studies

Text S2. Equation to estimate the risk of melioidosis and relative risk associated with each risk factor

Text S3. Questionnaire used for elicitation of expert opinion

Text S4. Melioidosis treatment and costing details

Table S3. Country-specific parameter inputs

Table S4. Incremental cost-effectiveness ratio (ICER) for different vaccination strategies (compared to the next most expensive strategy) by region.

Figure S2. Incremental cost-effectiveness ratio (ICER) for different vaccination strategies (compared to no vaccination) by region

Table S5. Results of the base case analysis (compared with no vaccination) by country/territory

Figure S3. Results from a sensitivity analysis assuming 50% vaccine protective efficacy.

Table S6. Results of all countries/territories from the sensitivity analysis, 50% vaccine protective efficacy.

**Table S1. Definition of clinical terms used in the main text**

| **Terms** | **Definitions** | **References** |
| --- | --- | --- |
| Acute melioidosis | Melioidosis with duration of symptoms less than 2 months prior to presentation | [1] |
| Acute melioidosis with complications | Acute melioidosis case with neuromelioidosis, persistant bacteraemia, or intensive care admission | [2] |
| Acute melioidosis without complications | Acute melioidosis case without neuromelioidosis, persistant bacteraemia, or intensive care admission | [2] |
| Chronic melioidosis | Melioidosis with duration of symptoms longer than 2 months prior to presentation | [1] |
| Chronic melioidosis with systemic illness | Chronic melioidosis case that is non-localised and distributed throughout the body including the bloodstream | [1] |
| Chronic melioidosis with non-systemic illness | Chronic melioidosis case that is indolent, non-fulminant and localised. |  |
| Risk of death | Probability that melioidosis patients die within one year after being diagnosed with melioidosis infection |  |

**References**

[1] Limmathurotsakul D, Koh GCKW,Peacock SJ, Currie BJ. Chronic melioidosis, relapse and latency. In: Melioidosis: A century of observation and research. Amsterdam: Elsevier B.V.; 2012:120-129.

[2] Lipsitz R, Garges S, Aurigemma R, Baccam P, Blaney DD, Cheng AC, et al. Workshop on Treatment of and Postexposure Prophylaxis for Burkholderia pseudomallei and B. mallei Infection, 2010. Emerg Infect Dis 2012; 18(12):1–11.

**Text S1. Literature used in the studies**

Text S1(a). List of 20 studies that reported the incidence of melioidosis and/or mortality rate (from Limmathurotsakul D, Golding N, Dance DAB, et al. Predicted global distribution of Burkholderia pseudomallei and burden of melioidosis. Nat Microbiol 2016; 1(Jan):1–5.)

1. Limmathurotsakul D, Wongratanacheewin S, Teerawattanasook N, et al. Increasing incidence of human melioidosis in northeast Thailand. Am J Trop Med Hyg **2010**; 82(6):1113–1117.
2. Liu X, Pang L, Sin SH, et al. Association of melioidosis incidence with rainfall and humidity, Singapore, 2003–2012. Emerg Infect Dis **2015**; 21(1):159–62.
3. Hanna JN, Humphreys JL, Brookes DL, Messina T, Raulli A. Melioidosis in north Queensland, 2000-2009. Commun Dis Intell Q Rep **2010**; 34(4):444–447.
4. Currie BJ, Ward L, Cheng AC. The epidemiology and clinical spectrum of melioidosis: 540 cases from the 20 year Darwin prospective study. PLoS NTD **2010**; 4(11):e900.
5. Parameswaran U, Baird RW, Ward LM, Currie BJ. Melioidosis at Royal Darwin Hospital in the big 2009-2010 wet season: comparison with the preceding 20 years. Med J Aust **2012**; 196(5):345-348.
6. Abdul Kadir KA, Sattayavani M, Pande K. Melioidosis: Antibiogram of cases in Brunei Darussalam. Brunei Int Med J **2014**; 10(1):19-24.
7. Vlieghe E, Kruy L, De Smet B, et al. Melioidosis, phnom penh, Cambodia. Emerg Infect Dis **2011**; 17(7):1289–92.
8. Dai D, Chen YS, Chen PS, Chen YL. Case cluster shifting and contaminant source as determinants of melioidosis in Taiwan. Trop Med Int Health **2012**; 17(8):1005–1013.
9. Chou DW, Chung KM, Chen CH, Cheung BM. Bacteremic melioidosis in southern Taiwan: clinical characteristics and outcome. J Formos Med Assoc **2007**; 106(12):1013–1022.
10. Quan HB, Li TY, Gao YY, Chen DX. Clinical features of diabetes mellitus cases complicated by Burkholderia pseudomallei septicemia. Genet Mol Res **2014**; 13(2):3108–16.
11. Jesudason MV, Anbarasu A, John TJ. Septicaemic melioidosis in a tertiary care hospital in south India. Indian J Med Res **2003**; 117:119–121.
12. Mohd Roslani AD, Tay ST, Puthucheary SD, Rukumani DV, Sam IC. Short report: Predictors of severe disease in melioidosis patients in Kuala Lumpur, Malaysia. Am J Trop Med Hyg **2014**; 91(6):1176–78.
13. Deris ZZ, Hasan H, Siti Suraiya MN. Clinical characteristics and outcomes of bacteraemic melioidosis in a teaching hospital in a northeastern state of Malaysia: a five-year review. J Infect Dev Ctries **2010**; 4(7):430-435.
14. Hassan MR, Pani SP, Peng NP, et al. Incidence, risk factors and clinical epidemiology of melioidosis: a complex socio-ecological emerging infectious disease in the Alor Setar region of Kedah, Malaysia. BMC Infect Dis **2010**; 10:302.
15. How SH, Ng TH, Jamalludin AR, et al. Pahang melioidosis registry. Med J Malaysia **2009**; 64(1):27–30.
16. Le Hello S, Currie BJ, Godoy D, et al. Melioidosis in New Caledonia. Emerg Infect Dis **2005**; 11(10):1607-9.
17. Warner JM, Pelowa DB, Currie BJ, Hirst RG. Melioidosis in a rural community of Western Province, Papua New Guinea. Trans R Soc Trop Med Hyg **2007**; 101(8):809–13.
18. Lo TJ, Ang LW, James L, Goh KT. Melioidosis in a tropical city state, Singapore. Emerg Infect Dis **2009**; 15(10):1645–7.
19. Bhengsri S, Baggett HC, Jorakate P, et al. Incidence of bacteremic melioidosis in eastern and northeastern Thailand. Am J Trop Med Hyg **2011**; 85(1):117-20.
20. Phuong DM, Trung TT, Breitbach K, et al. Clinical and microbiological features of melioidosis in northern Vietnam. Trans R Soc Trop Med Hyg **2008**; 102(Suppl 1):S30–36.

Text S1(b). List of 11 studies that reported the melioidosis cases related to either diabetes, chronic renal disease, or age group used in the relative risk estimates. (from Limmathurotsakul D, Golding N, Dance DAB, et al. Predicted global distribution of Burkholderia pseudomallei and burden of melioidosis. Nat Microbiol 2016; 1(Jan):1–5.)

1. Limmathurotsakul D, Wongratanacheewin S, Teerawattanasook N, et al. Increasing incidence of human melioidosis in northeast Thailand. *Am J Trop Med Hyg.* 2010;**82**(6):1113–1117.
2. Hanna JN, Humphreys JL, Brookes DL, Messina T, Raulli A. Melioidosis in north Queensland, 2000-2009. *Commun Dis Intell Q Rep* 2010;**34**(4):444–447.
3. Currie BJ, Ward L, Cheng AC. The epidemiology and clinical spectrum of melioidosis: 540 cases from the 20 year Darwin prospective study. *PLoS NTD* 2010; **4**(11):e900.
4. Parameswaran U, Baird RW, Ward LM, Currie BJ. Melioidosis at Royal Darwin Hospital in the big 2009-2010 wet season: comparison with the preceding 20 years. *Med J Aust* 2012;**196**(5):345-348.
5. Vlieghe E, Kruy L, De Smet B, et al. Melioidosis, Phnom Penh, Cambodia. *Emerg Infect Dis* 2011;**17**(7):1289–92.
6. Chou DW, Chung KM, Chen CH, Cheung BM. Bacteremic melioidosis in southern Taiwan: clinical characteristics and outcome. *J Formos Med Assoc* 2007;**106**(12):1013–1022.
7. Mohd Roslani AD, Tay ST, Puthucheary SD, Rukumani DV, Sam IC. Short report: Predictors of severe disease in melioidosis patients in Kuala Lumpur, Malaysia. *Am J Trop Med Hyg* 2014;**91**(6):1176–78.
8. Deris ZZ, Hasan H, Siti Suraiya MN. Clinical characteristics and outcomes of bacteraemic melioidosis in a teaching hospital in a northeastern state of Malaysia: a five-year review. *J Infect Dev Ctries* 2010;**4**(7):430-435.
9. Hassan MR, Pani SP, Peng NP, et al. Incidence, risk factors and clinical epidemiology of melioidosis: a complex socio-ecological emerging infectious disease in the Alor Setar region of Kedah, Malaysia. *BMC Infect Dis* 2010;**10**:302.
10. Lo TJ, Ang LW, James L, Goh KT. Melioidosis in a tropical city state, Singapore. *Emerg Infect Dis* 2009;**15**(10):1645–7.
11. Phuong DM, Trung TT, Breitbach K, et al. Clinical and microbiological features of melioidosis in northern Vietnam. *Trans R Soc Trop Med Hyg* 2008;**102(**Suppl 1):S30–36.

Text S1(c). List of 5 studies that reported cost per bed day for both ICU and non-ICU stays, from a rapid review of literature.

1. Acharya SP. Critical care medicine in Nepal: where are we? Int Health. **2013**; 5(Sep):92–5.

2. Baker T. Critical care in low-income countries. Trop Med Int Heal. **2009**; 14(2):143–8.

3. Kumar P, Jithesh V, Gupta S. A comparative cost analysis of polytrauma and neurosurgery Intensive Care Units at an apex trauma care facility in India. Indian J Crit Care Med. **2016**; 20(7):398.

3. Karabatsou D, Tsironi M, Tsigou E, Boutzouka E, Katsoulas T, Baltopoulos G. Variable cost of ICU care, a micro-costing analysis. Intensive Crit Care Nurs. **2016**; 35:66–73.

4. Chin-Yee N, D’Egidio G, Thavorn K, Heyland D, Kyeremanteng K. Cost analysis of the very elderly admitted to intensive care units. Crit Care. **2017**; 21(1):109.

5. Lindemark F, Haaland ØA, Kvåle R, Flaatten H, Norheim OF, Johansson KA. Costs and expected gain in lifetime health from intensive care versus general ward care of 30,712 individual patients: a distribution-weighted cost-effectiveness analysis. Crit Care. **2017**; 21(1):220.

**Figure S1. Detailed results for the Bayesian synthesis of 12 observational studies.**

Figure S1(a). Predicted number of melioidosis cases for each subgroup based on a Bayesian synthesis of 12 observational studies (red boxes) compared to actual reported cases in each study (black boxes). Study numbers refer to the list in Text S1(a), with the sample size listed. Abbreviations: N = no risk factors, A = age > 45 years, D = diabetes, R = chronic renal disease, etc.

Figure S1(b). Estimated probability density function of relative risk of melioidosis associated with each risk factor (age >45 years, diabetes, and chronic renal disease) compared to no risk factor.


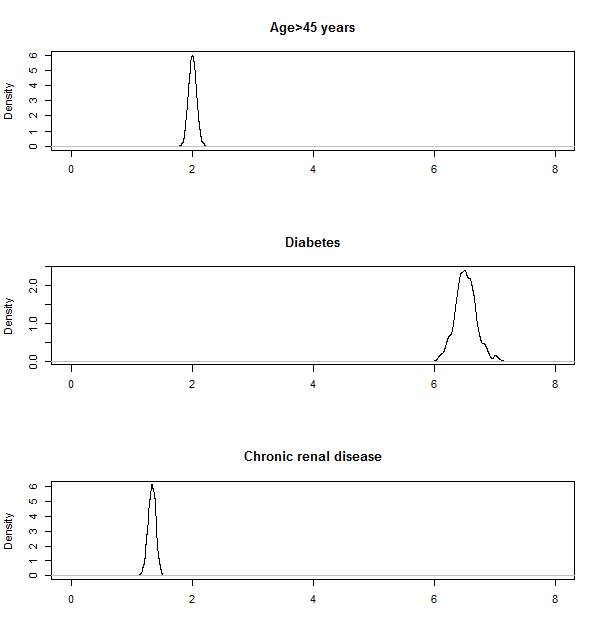


| **Risk factors** | **Mean** | **95% uncertainty interval** |
| --- | --- | --- |
| Age >45 years | 2.01 | (1.88, 2.16) |
| Diabetes | 6.49 | (6.10, 6.90) |
| Chronic renal disease | 1.33 | (1.21, 1.46) |

Figure S1(c). Correlation between risk factors

**
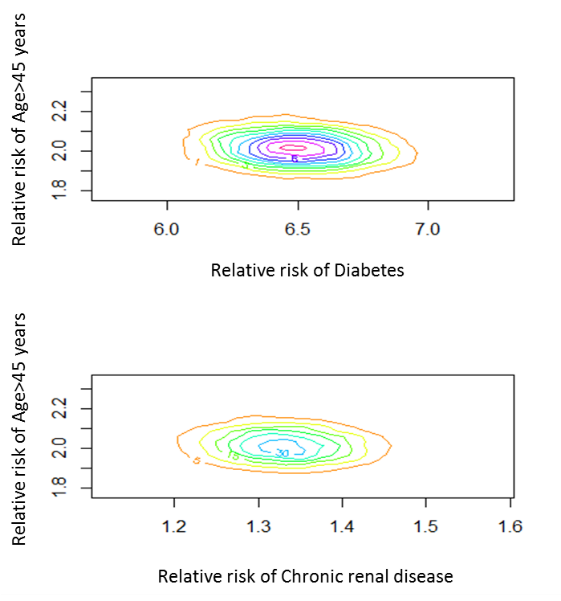
**

Table S2. Extracted data about the number of melioiosis cases by subgroup determined by presence of risk factors, from the observational studies reviewed.

| **Study** | **Authors** | **Year** | **Country** | **Total** | ***N** | **A** | **D** | **R** | **AD** | **AR** | **DR** | **ADR** |
| --- | --- | --- | --- | --- | --- | --- | --- | --- | --- | --- | --- | --- |
| 1 | Limmathurotsakul et al. | 2010 | Thailand | 2217 | 470 | 734 | 311 | 50 | 487 | 79 | 33 | 52 |
| 2 | Currie et al. | 2010 | Australia | 364 | 84 | 125 | 49 | 8 | 73 | 12 | 5 | 7 |
| 3 | Hanna et al. | 2010 | Australia | 173 | 45 | 31 | 45 | 6 | 31 | 4 | 6 | 4 |
| 4 | Mohd Ros et al. | 2014 | Malaysia | 85 | 7 | 16 | 15 | 1 | 33 | 3 | 3 | 7 |
| 5 | Deris et al. | 2010 | Malaysia | 27 | 5 | 2 | 4 | 1 | 12 | 0 | 1 | 2 |
| 6 | Parameswaran et al. | 2012 | Australia | 540 | 288 | n/a | 187 | 39 | n/a | n/a | 26 | n/a |
| 7 | Parameswaran et al. | 2012 | Australia | 91 | 43 | n/a | 41 | 4 | n/a | n/a | 3 | n/a |
| 8 | Chou et al. | 2007 | Taiwan | 30 | 12 | n/a | 12 | 3 | n/a | n/a | 3 | n/a |
| 9 | Hassan et al. | 2010 | Malaysia | 145 | 57 | n/a | 74 | 6 | n/a | n/a | 8 | n/a |
| 10 | Lo et al. | 2009 | Singapore | 693 | 397 | n/a | 226 | 45 | n/a | n/a | 25 | n/a |
| 11 | Phuong et al. | 2008 | Vietnam | 40 | 28 | n/a | 9 | 2 | n/a | n/a | 1 | n/a |
| 12 | Vlieghe et al. | 2011 | Cambodia | 51 | 10 | 9 | 17 | n/a | 15 | n/a | n/a | n/a |

*Single and combined risk groups are N: no risk factors, A: age over either 40, 45, 50, or 55 (represent the cut off at age 45), D: diabetes, R: chronic renal disease, AD: diabetes who age over 45, AR: chronic renal disease who age over 45, DR: diabetes and chronic renal disease, ADR: diabetes and chronic renal disease who age over 45.

Text S2. Equation to estimate the risk of melioidosis and relative risk associated with each risk factor

Number of Melioidosis Cases = *r.P + r_A_.P_A_ + r_D_.P_D_ + r_R_.P_R_ + r_AD_.P_AD_ + r_AR_.P_AR_ + r_DR_.P_DR_ + r_ADR_.P_ADR_*

| **Symbol** | **Definition** |
| --- | --- |
| *r* | risk of melioidosis for population aged under 45 without any risk factors (Baseline risk) |
| *r_A_* | risk of melioidosis for population aged over 45 without any risk factors |
| *r_D_* | risk of melioidosis for population aged under 45 with diabetes |
| *r_R_* | risk of melioidosis for population aged under 45 with chronic renal disease |
| *r_AD_* | risk of melioidosis for population aged over 45 with diabetes |
| *r_AR_* | risk of melioidosis for population aged over 45 with chronic renal disease |
| *r_DR_* | risk of melioidosis for population aged under 45 with diabetes and chronic renal disease |
| *r_ADR_* | risk of melioidosis for population aged over 45 with diabetes and chronic renal disease |
| *P* | Population at risk aged under 45 without any risk factors |
| *P_A_* | Population at risk aged over 45 without any risk factors |
| *P_D_* | Population at risk aged under 45 with diabetes |
| *P_R_* | Population at risk aged under 45 with chronic renal disease |
| *P_AD_* | Population at risk aged over 45 with diabetes |
| *P_AR_* | Population at risk aged over 45 with chronic renal disease |
| *P_DR_* | Population at risk aged under 45 with diabetes and chronic renal disease |
| *P_ADR_* | Population at risk aged over 45 with diabetes and chronic renal disease |

**Text S3. Questionnaire used for elicitation of expert opinion.**

**Introduction**

The London School of Hygiene & Tropical Medicine and the University of Exeter are conducting a cost-effectiveness analysis of a potential vaccine against *B. pseudomallei* to inform future investment cases for developing such a vaccine. The analysis is informed by a mathematical model simulating the impact of the hypothetical melioidosis vaccine intervention in 83 countries mapped in a recent study (Limmathurotsakul D et al. Nat Microbiol. 2016 Jan 1;1(1). pii: 15008). In the model, patients presenting to hospital with melioidosis are classified into four conditions based on clinical acquisition and presentation: i) acute with complications, ii) acute without complications, iii) chronic with systemic illness, and iv) chronic with non-systemic illness. Please see definitions for these conditions below. Patients with different conditions are assumed to have different risks of death.

There is limited information in the published literature about the proportion of patients with each of these conditions, their risk of death among each of these conditions, and their risk of ICU attendance proportion of all acute cases with complications. Hence, we are conducting an expert elicitation exercise to estimate these key model inputs. As an expert in this field, we would like to ask you to elicit the value of these parameters to be used in the model.

Note that we would like to acknowledge by name all experts who contributed to this exercise in the Acknowledgments section of any subsequent publication, but individual elicited values will not be associated with any named person.

**Definitions**

References for these definitions are: (i) Limmathurotsakul D. et al. 2012, Chronic melioidosis, relapse and latency In Ketheesan N. Melioidosis-A Century of Observation and Research, 2012, (ii) Lipsitz R et al. 2012.

| **Terms** | **Definitions** |
| --- | --- |
| Acute melioidosis | Melioidosis with duration of symptoms for less than 2 months prior to presentation. |
| Acute melioidosis with complications | Acute melioidosis cases with neuromelioidosis, persistant bacteraemia, or admitted ICU. |
| Acute melioidosis without complications | Acute melioidosis cases without neuromelioidosis, persistant bacteraemia, or admitted ICU. |
| Chronic melioidosis | Melioidosis with duration of symptoms longer than 2 months prior to presentation. |
| Chronic melioidosis with systemic illness | Chronic melioidosis cases with evidence of non-localised and distributed throughout the body including bloodstream. |
| Chronic melioidosis with non-systemic illness | Chronic melioidosis with indolent, non-fulminant and localised. |

The elicitation exercise begins on the next page.

**Please fill in the number in the box below**

1. **Estimate the proportion of all patients presenting to hospital with melioidosis that have each condition below:**

|  | **Acute with complications** | **Acute without complications** | **Chronic with systemic illness** | **Chronic without systemic illness** |
| --- | --- | --- | --- | --- |
| **High income country**  **(eg. Australia)** |  |  |  |  |
| **Middle income country**  **(eg. Thailand)** |  |  |  |  |
| **Low income country**  **(eg. Guinea)** |  |  |  |  |

**For example:** If you think that in a high income country, of all patients presenting to hospital with melioidosis, 20% are acute with complications, 30% are acute without complications, 20% are chronic with systematic illness and 30% are chronic without systemic illness, then you would write 20%, 30%, 20% and 30% in the four boxes on the first row.

1. **Estimate the risk of death of each of the melioidosis conditions**

| **Items** | **Best estimate** |
| --- | --- |
| Of all ***acute patients with complications*** presenting to hospital, what is the proportion of patients who die from melioidosis? |  |
| Of all ***acute patients without complications*** presenting to hospital, what is the proportion of patients who die from melioidosis? |  |
| Of all ***chronic patients with systemic illness*** presenting to hospital, what is the proportion of patients who die from melioidosis? |  |
| Of all ***chronic patients without systemic illness*** presenting to hospital, what is the proportion of patients who die from melioidosis? |  |

1. **Estimate the proportion of ICU admission amongst acute patients with complications**

| **Items** | **Best estimate** |
| --- | --- |
| Of all ***acute patients with complications*** presenting to hospital, what is the proportion of patients admitted to **ICU**? |  |

**Text S4. Melioidosis treatment and costing details**

The costs of melioidosis treatment were estimated by micro-costing. Melioidosis cases were generally assumed to be treated with regimens in compliance with treatment guidelines [1]. This splits treatment protocols into two phases: 10-14 day intensive phase and 84-140 day eradication phase. The duration of the two phases and the length of hospitalisation within the two phases was obtained from analysing patient data from a hospital in India (Additional file 2; manuscript in preparation). We assumed these durations were the same across geographies, although there are some minor differences.

During the intensive treatment period, acute cases with severe complications (e.g. septic shock) receive intravenous meropenem while acute cases without complications and chronic patients receive intravenous ceftazidime. However, ceftazidime was assumed to be used for acute cases with complications in low-income countries given that meropenem is often unavailable or unaffordable in these countries. During the eradication phase, patients receive oral trimethoprim/sulfamethoxazole (co-trimoxazole) as first line treatment and co-amoxiclav as second line therapy. We assumed 95% of patients received first line treatment. Children (age under 15) and adults (age over 15) were assumed to be treated with different doses due to differences in weight [1].

The costs of antibiotics were assumed to be different in low, middle and high income geographies [2]. Antibiotic costs for high and middle-income geographies was estimated from UK and Thai data respectively [3–5]. Because of the lack of information about antibiotic costs in low-income geographies, we assumed two-thirds of the costs in middle-income geographies. Cost of hospitalisation (by bed day) in each geography was obtained from the WHO-CHOICE database [6]. The additional cost of a bed-day in an intensive care ward was estimated for each geography income level by the average ratio of cost per bed day between non-ICU and ICU obtained from a rapid review of literature. (Supplementary material Appendix J) All costs were inflated to the year 2016 [7]. All geography-specific epidemiological and economic parameter inputs are shown in Supplementary material Appendix F.

**References**

**[1]** Lipsitz R, Garges S, Aurigemma R, et al. Workshop on Treatment of and Postexposure Prophylaxis for Burkholderia pseudomallei and B. mallei Infection, 2010. Emerg Infect Dis **2012**; 18(12):1–11.

**[2]** The World Bank, World Development Indicators (2016). GNI per capita, Atlas method [Data file]. Retrieved from <http://data.worldbank.org/indicator/NY.GNP.PCAP.CD?locations=XM-XD-XT-XN>.

**[3]** Drug Medical Supply and Information Center (DMSIC), Ministry of Public Health Thailand [online]. Available at: <http://dmsic.moph.go.th/dmsic/force_down.php?f_id=716>. Accessed 2017 August.

**[4]** Joint Formulary Committee (2017), British national formulary. [online]

Available at: <https://bnf.nice.org.uk/>. Accessed 2017 August.

**[5]** Drug Tariff (2017), National Health Services (NHS) UK. [online]

Available at: https://www.nhsbsa.nhs.uk/pharmacies-gp-practices-and-appliance-contractors/drug-tariff. Accessed 2017 August.

**[6]** WHO-CHOICE unit cost estimates for service delivery, The World Health Organisation (WHO) [online] Available at: <http://www.who.int/choice/cost-effectiveness/inputs/health_service/en/>

**[7]** The World Bank, World Development Indicators (2016). Consumer price index [Data file]. Retrieved from <https://data.worldbank.org/indicator/FP.CPI.TOTL.ZG?end=2016&start=2007>.

**Table S3. Country-specific parameter inputs**

Table S3(a). Country-specific epidemiological parameters.

| Country | Overall Incidence per 100,000 | | Incidence per 100,000 in environmental suitability area | | Population at risk | Population in predicted  environmental suitability area | Diabetes  prevalence | Renal  disease prevalence |
| --- | --- | --- | --- | --- | --- | --- | --- | --- |
| Angola | 0.116 | | 2.84 | | 25,021,974 | 1,022,143 | 0.0291 | 0.076 |
| Argentina | 0.041 | | 1.77 | | 43,416,755 | 1,017,517 | 0.0557 | 0.121 |
| Australia | 0.627 | | 11.24 | | 23,781,169 | 1,325,901 | 0.0955 | 0.0814 |
| Bangladesh | 10.516 | | 12.36 | | 160,995,642 | 137,042,130 | 0.0711 | 0.0676 |
| Benin | 8.447 | | 15.46 | | 10,879,829 | 5,946,347 | 0.0168 | 0.076 |
| Bhutan | 1.678 | | 11.75 | | 774,830 | 110,626 | 0.0573 | 0.0676 |
| Bolivia | 0.121 | | 3.49 | | 10,724,705 | 372,529 | 0.0689 | 0.121 |
| Brazil | 0.420 | | 3.97 | | 207,847,528 | 21,966,596 | 0.1052 | 0.121 |
| Brunei | 6.853 | | 7.93 | | 423,188 | 365,876 | 0.1044 | 0.1173 |
| Burkina Faso | 3.463 | | 8.56 | | 18,105,570 | 7,323,307 | 0.0295 | 0·076 |
| Cambodia | 13.372 | | 17.24 | | 15,577,899 | 12,083,816 | 0.0296 | 0·086 |
| Cameroon | 2.313 | | 7.36 | | 23,344,179 | 7,337,382 | 0.0615 | 0·076 |
| Central African Republic | 2.898 | | 7.65 | | 4,900,274 | 1,855,173 | 0.0305 | 0·076 |
| Chad | 2.857 | | 7.75 | | 14,037,472 | 5,176,835 | 0.0361 | 0·076 |
| China | 0.523 | | 6.60 | | 1,371,220,000 | 108,655,616 | 0.0882 | 0.1006 |
| Colombia | 0.326 | | 4.01 | | 48,228,704 | 3,911,599 | 0.0726 | 0.121 |
| Congo, Dem. Rep. | 0.287 | | 4.02 | | 77,266,814 | 5,524,442 | 0.0303 | 0.076 |
| Congo, Rep. | 5.671 | | 14.18 | | 4,620,330 | 1,847,393 | 0.0552 | 0.076 |
| Costa Rica | 0.333 | | 3.59 | | 4,807,850 | 446,210 | 0.0881 | 0.121 |
| Cote d'Ivoire | 5.039 | | 11.22 | | 22,701,556 | 10,195,769 | 0.0493 | 0.076 |
| Cuba | 0.176 | | 2.47 | | 11,389,562 | 808,792 | 0.0858 | 0.121 |
| El Salvador | 1.861 | | 5.72 | | 6,126,583 | 1,993,787 | 0.0988 | 0.121 |
| Equatorial Guinea | 0.710 | | 2.77 | | 845,060 | 216,569 | 0.0421 | 0.076 |
| Eritrea | 0.413 | | 4.20 | | 6,540,000 | 642,872 | 0.0341 | 0.076 |
| Ethiopia | 0.263 | | 4.10 | | 99,390,750 | 6,362,580 | 0.0332 | 0.076 |
| Fiji | 0.448 | | 2.76 | | 892,145 | 144,975 | 0.1086 | 0.1173 |
| Gabon | 2.608 | | 6.89 | | 1,725,292 | 653,568 | 0.1019 | 0.076 |
| Gambia, The | 0.402 | | 3.33 | | 1,990,924 | 240,094 | 0.0197 | 0.076 |
| Ghana | 1.419 | | 6.39 | | 27,409,893 | 6,088,033 | 0.0316 | 0.076 |
| Guatemala | 0.404 | | 4.63 | | 16,342,897 | 1,425,082 | 0.0993 | 0.121 |
| Guinea | 10.881 | | 14.50 | | 12,608,590 | 9,459,923 | 0.0436 | 0.076 |
| Guinea-Bissau | 5.422 | | 9.36 | | 1,844,325 | 1,068,314 | 0.0300 | 0.076 |
| Guyana | 1.564 | | 4.60 | | 767,085 | 260,835 | 0.1608 | 0.121 |
| Haiti | 0.224 | | 3.20 | | 10,711,067 | 750,572 | 0.0666 | 0.121 |
| Honduras | 1.065 | | 5.89 | | 8,075,060 | 1,461,303 | 0.0714 | 0.121 |
| Hong Kong | 0.917 | | 2.38 | | 7,305,700 | 2,815,656 | 0.0761 | 0.1006 |
| India | 4.005 | | 9.05 | | 1,311,050,527 | 580,113,723 | 0.0901 | 0.0676 |
| Indonesia | 7.780 | | 15.28 | | 257,563,815 | 131,111,720 | 0.0514 | 0.1173 |
| Iran | 0.019 | | 1.31 | | 79,109,272 | 1,142,355 | 0.1059 | 0.1168 |
| Iraq | 0.058 | | 2.16 | | 36,423,395 | 970,440 | 0.0971 | 0.1168 |
| Kenya | 0.217 | | 4.32 | | 46,050,302 | 2,317,436 | 0.0466 | 0.076 |
| Lao PDR | 6.175 | | 11.32 | | 6,802,023 | 3,711,102 | 0.0514 | 0.086 |
| Liberia | 9.881 | | 13.79 | | 4,503,438 | 3,226,827 | 0.0312 | 0.076 |
| Madagascar | 3.631 | | 10.75 | | 24,235,390 | 8,187,337 | 0.0509 | 0.076 |
| Malawi | 1.284 | | 5.64 | | 17,215,232 | 3,916,621 | 0.0563 | 0.076 |
| Malaysia | 5.776 | | 8.58 | | 30,331,007 | 20,415,758 | 0.1202 | 0.086 |
| Mali | 3.296 | | 8.14 | | 17,599,694 | 7,125,305 | 0.0167 | 0.076 |
| Mauritania | 0.688 | | 3.67 | | 4,067,564 | 762,563 | 0.0364 | 0.076 |
| Mauritius | 0.396 | | 1.85 | | 1,262,605 | 270,794 | 0.1476 | 0.076 |
| Mexico | 0.433 | | 3.80 | | 127,017,224 | 14,486,081 | 0.1559 | 0.121 |
| Mozambique | 0.851 | | 4.95 | | 27,977,863 | 4,812,761 | 0.0314 | 0.076 |
| Myanmar | 11.591 | | 18.03 | | 53,897,154 | 34,639,423 | 0.0589 | 0.086 |
| Nepal | 3.205 | | 9.59 | | 28,513,700 | 9,529,719 | 0.0358 | 0.676 |
| Nicaragua | 1.069 | | 4.08 | | 6,082,032 | 1,592,084 | 0.1158 | 0.121 |
| Niger | 1.849 | | 4.71 | | 19,899,120 | 7,815,890 | 0.0415 | 0.076 |
| Nigeria | 7.399 | | 13.74 | | 182,201,962 | 98,150,456 | 0.0483 | 0.076 |
| Oman | 0.134 | | 1.25 | | 4,490,541 | 479,760 | 0.1016 | 0.1168 |
| Pakistan | 0.234 | | 3.18 | | 188,924,874 | 13,919,904 | 0.0789 | 0.0676 |
| Panama | 1.705 | | 5.22 | | 3,929,141 | 1,283,096 | 0.0859 | 0.121 |
| Papua New Guinea | 1.693 | | 7.40 | | 7,619,321 | 1,743,547 | 0.0649 | 0.1173 |
| Paraguay | 0.196 | | 3.57 | | 6,639,123 | 363,806 | 0.0681 | 0.121 |
| Peru | 0.124 | | 4.71 | | 31,376,670 | 827,631 | 0.0681 | 0.121 |
| Philippines | 9.053 | | 13.45 | | 100,699,395 | 67,756,366 | 0.0965 | 0.1173 |
| Saudi Arabia | 0.165 | | 1.30 | | 31,540,372 | 3,990,536 | 0.2338 | 0.1168 |
| Senegal | 0.397 | | 4.44 | | 15,129,273 | 1,352,038 | 0.0326 | 0.076 |
| Sierra Leone | 9.298 | | 13.60 | | 6,453,184 | 4,412,594 | 0.0307 | 0.076 |
| Singapore | 4.986 | | 6.12 | | 5,535,002 | 4,511,882 | 0.1045 | 0.086 |
| Somalia | 0.658 | | 4.67 | | 10,787,104 | 1,520,015 | 0.0387 | 0.076 |
| South Africa | 0.051 | | 2.46 | | 54,956,920 | 1,140,164 | 0.0704 | 0.076 |
| South Sudan | 0.316 | | 3.18 | | 12,339,812 | 1,227,321 | 0.0912 | 0.076 |
| Sri Lanka | 8.972 | | 16.37 | | 20,966,000 | 11,487,842 | 0.0748 | 0.0676 |
| Sudan | 0.154 | | 3.04 | | 40,234,882 | 2,038,914 | 0.0912 | 0.076 |
| Suriname | 2.578 | | 5.28 | | 542,975 | 265,187 | 0.1167 | 0.121 |
| Tanzania | 0.574 | | 6.42 | | 53,470,420 | 4,778,812 | 0.0281 | 0.076 |
| Thailand | 11.142 | | 14.80 | | 67,959,359 | 51,170,471 | 0.0626 | 0.086 |
| Timor-Leste | 0.803 | | 3.94 | | 1,245,015 | 253,732 | 0.0638 | 0.1173 |
| Togo | 2.149 | | 6.89 | | 7,304,578 | 2,280,150 | 0.0521 | 0.076 |
| Uganda | 0.077 | | 2.26 | | 39,032,383 | 1,330,139 | 0.0285 | 0.076 |
| Venezuela, RB | 0.331 | | 4.00 | | 31,108,083 | 2,572,384 | 0.1039 | 0.121 |
| Vietnam | 11.374 | | 16.31 | | 91,703,800 | 63,967,623 | 0.0578 | 0.086 |
| Yemen, Rep | 0.369 | | 2.81 | | 26,832,215 | 3,523,919 | 0.0922 | 0.1168 |
| Zambia | 0.691 | | 5.09 | | 16,211,767 | 2,202,315 | 0.0513 | 0.076 |
| Zimbabwe | 0.045 | | 1.91 | | 15,602,751 | 365,979 | 0.0975 | 0.076 |
|  |  |  | |  | |  |  |  |

Table S3(b). Country-specific economic parameters - adults (15 years and over).

| **Country** | **Income level** | **Antibiotic costs** | | | | **Treatment costs** | | **Productivity**  **costs** |
| --- | --- | --- | --- | --- | --- | --- | --- | --- |
|  |  | **Meropenem** | **Ceftazidime** | **Trimethoprim/**  **Sulfamethoxazole** | **Co-amoxiclav** | **Inpatient bed day** | **ICU bed day** |  |
| Angola | L | n/a | 5**.**35 | 0**.**06 | 2**.**11 | 95**.**79 | 1,272**.**32 | 11**.**23 |
| Argentina | M | 79**.**30 | 5**.**35 | 0**.**06 | 2**.**11 | 155**.**04 | 1,228**.**06 | 36**.**77 |
| Australia | H | 62**.**37 | 43**.**39 | 1**.**21 | 1**.**06 | 807**.**86 | 2,067**.**78 | 154**.**22 |
| Bangladesh | L | n/a | 5**.**35 | 0**.**06 | 2.11 | 6**.**22 | 82**.**67 | 3**.**32 |
| Benin | L | n/a | 5**.**35 | 0**.**06 | 2.11 | 6**.**94 | 92**.**19 | 2**.**13 |
| Bhutan | L | n/a | 5**.**35 | 0**.**06 | 2**.**11 | 28**.**66 | 380**.**68 | 6**.**93 |
| Bolivia | L | n/a | 5**.**35 | 0**.**06 | 2**.**11 | 24**.**32 | 323**.**07 | 8**.**47 |
| Brazil | M | 79**.**30 | 5**.**35 | 0**.**06 | 2**.**11 | 2776 | 219**.**85 | 23**.**38 |
| Brunei | H | 62**.**37 | 43**.**39 | 1**.**21 | 1**.**06 | 544**.**33 | 1,393**.**23 | 100**.**23 |
| Burkina Faso | L | n/a | 5**.**35 | 0**.**06 | 2**.**11 | 5**.**38 | 71**.**44 | 1**.**68 |
| Cambodia | L | n/a | 5**.**35 | 0**.**06 | 2**.**11 | 8**.**97 | 119**.**10 | 3**.**17 |
| Cameroon | L | n/a | 5**.**35 | 0**.**06 | 2**.**11 | 11**.**75 | 156**.**04 | 3**.**42 |
| Central African Republic | L | n/a | 5**.**35 | 0**.**06 | 2**.**11 | 6**.**16 | 81**.**78 | 0**.**84 |
| Chad | L | n/a | 5**.**35 | 0**.**06 | 2**.**11 | 7**.**39 | 98**.**17 | 2**.**12 |
| China | M | 79**.**30 | 5**.**35 | 0**.**06 | 2**.**11 | 39**.**58 | 313**.**54 | 21**.**70 |
| Colombia | M | 79**.**30 | 5**.**35 | 0**.**06 | 2**.**11 | 72**.**88 | 577**.**28 | 16**.**58 |
| Congo, Dem. Rep. | L | n/a | 5.35 | 0**.**06 | 2**.**11 | 1**.**59 | 21**.**18 | 1**.**25 |
| Congo, Rep. | L | n/a | 5**.**35 | 0**.**06 | 2**.**11 | 35**.**15 | 466**.**82 | 5**.**07 |
| Costa Rica | M | 79**.**30 | 5**.**35 | 0**.**06 | 2**.**11 | 108**.**76 | 861**.**48 | 29**.**10 |
| Cote d'Ivoire | L | n/a | 5**.**35 | 0**.**06 | 2**.**11 | 9**.**91 | 131**.**59 | 3**.**83 |
| Cuba | M | 79**.**30 | 5**.**35 | 0**.**06 | 2**.**11 | 69**.**19 | 548**.**01 | 18**.**59 |
| El Salvador | L | n/a | 5**.**35 | 0**.**06 | 2**.**11 | 40**.**51 | 538**.**11 | 11**.**55 |
| Equatorial Guinea | M | 79**.**30 | 5**.**35 | 0**.**06 | 2**.**11 | 519**.**42 | 4,114**.**27 | 30**.**45 |
| Eritrea | L | n/a | 5**.**35 | 0**.**06 | 2**.**11 | 2**.**97 | 39**.**48 | 1**.**49 |
| Ethiopia | L | n/a | 5**.**35 | 0**.**06 | 2**.**11 | 7**.**33 | 97**.**36 | 1**.**70 |
| Fiji | M | 79**.**30 | 5**.**35 | 0**.**06 | 2**.**11 | 50**.**49 | 399**.**95 | 13**.**46 |
| Gabon | M | 79**.**30 | 5**.**35 | 0**.**06 | 2**.**11 | 133**.**22 | 1,055**.**23 | 22**.**76 |
| Gambia | L | n/a | 5**.**35 | 0**.**06 | 2.11 | 5**.**23 | 69**.**49 | 1**.**21 |
| Ghana | L | n/a | 5**.**35 | 0**.**06 | 2**.**11 | 14**.**03 | 186**.**39 | 3**.**78 |
| Guatemala | L | n/a | 5**.**35 | 0**.**06 | 2**.**11 | 36**.**56 | 485**.**57 | 10.69 |
| Guinea | L | n/a | 5**.**35 | 0**.**06 | 2**.**11 | 6**.**88 | 91**.**38 | 1**.**45 |
| Guinea-Bissau | L | n/a | 5**.**35 | 0**.**06 | 2**·**11 | 2**.**08 | 27**.**59 | 1**.**57 |
| Guyana | M | 79**.**30 | 5**.**35 | 0**.**06 | 2**.**11 | 15**.**51 | 122**.**83 | 11**.**30 |
| Haiti | L | n/a | 5**.**35 | 0**.**06 | 2**.**11 | 7**.**45 | 98**.**97 | 2**.**27 |
| Honduras | L | n/a | 5**.**35 | 0**.**06 | 2**.**11 | 26**.**33 | 349**.**75 | 6**.**83 |
| Hong Kong | H | 62**.**37 | 43**.**39 | 1**.**21 | 1**.**06 | 679**.**00 | 1,737**.**93 | 116**.**15 |
| India | L | n/a | 5**.**35 | 0**.**06 | 2**.**11 | 17**.**54 | 233**.**00 | 4**.**33 |
| Indonesia | L | n/a | 5**.**35 | 0**.**06 | 2**.**11 | 31**.**08 | 412**.**81 | 9**.**16 |
| Iran | M | 79**.**30 | 5**.**35 | 0**.**06 | 2**.**11 | 216**.**69 | 1,716**.**38 | 14**.**90 |
| Iraq | M | 79**.**30 | 5**.**35 | 0**.**06 | 2**.**11 | 41**.**10 | 325**.**54 | 12**.**67 |
| Kenya | L | n/a | 5**.**35 | 0**.**06 | 2**.**11 | 12**.**17 | 161**.**58 | 3**.**77 |
| Lao | L | n/a | 5**.**35 | 0**.**06 | 2**.**11 | 9**.**30 | 123**.**56 | 4**.**96 |
| Liberia | L | n/a | 5**.**35 | 0**.**06 | 2**.**11 | 2**.**46 | 32**.**65 | 1**.**25 |
| Madagascar | L | n/a | 5**.**35 | 0**.**06 | 2**.**11 | 6**.**02 | 80**.**01 | 1**.**13 |
| Malawi | L | n/a | 5**.**35 | 0**.**06 | 2**.**11 | 5**.**49 | 72**.**91 | 1**.**04 |
| Malaysia | M | 79**.**30 | 5**.**35 | 0**.**06 | 2**.**11 | 110**.**06 | 871**.**74 | 26**.**74 |
| Mali | L | n/a | 5**.**35 | 0**.**06 | 2**.**11 | 5**.**84 | 77**.**55 | 2**.**04 |
| Mauritania | L | n/a | 5**.**35 | 0**.**06 | 2**.**11 | 11**.**22 | 149**.**01 | 3**.**75 |
| Mauritius | M | 79**.**30 | 5**.**35 | 0**.**06 | 2**.**11 | 108**.**51 | 859**.**47 | 24**.**96 |
| Mexico | M | 79**.**30 | 5**.**35 | 0**.**06 | 2**.**11 | 155**.**27 | 1,229**.**86 | 24**.**67 |
| Mozambique | L | n/a | 5**.**35 | 0**.**06 | 2**.**11 | 4**.**54 | 60**.**33 | 1**.**44 |
| Myanmar | L | n/a | 5**.**35 | 0**.**06 | 2**.**11 | 6**.**17 | 82**.**01 | 3**.**30 |
| Nepal | L | n/a | 5**.**35 | 0**.**06 | 2**.**11 | 5**.**61 | 74**.**57 | 2**.**00 |
| Nicaragua | L | n/a | 5**.**35 | 0**.**06 | 2**.**11 | 16**.**14 | 214**.**38 | 5**.**71 |
| Niger | L | n/a | 5**.**35 | 0**.**06 | 2**·**11 | 2**.**69 | 35**.**70 | 0**.**98 |
| Nigeria | L | n/a | 5**.**35 | 0**.**06 | 2**.**11 | 24**.**56 | 326**.**26 | 7**.**23 |
| Oman | H | 62**.**37 | 43**.**39 | 1**.**21 | 1**.**06 | 361**.**13 | 924**.**34 | 42**.**83 |
| Pakistan | L | n/a | 5**.**35 | 0**.**06 | 2.11 | 14**.**54 | 193**.**19 | 3**.**91 |
| Panama | M | 79**.**30 | 5**.**35 | 0**.**06 | 2**.**11 | 101**.**67 | 805**.**35 | 36**.**33 |
| Papua New Guinea | L | n/a | 5**.**35 | 0**.**06 | 2**.**11 | 15**.**07 | 200**.**12 | 6**.**21 |
| Paraguay | M | 79**.**30 | 5**.**35 | 0**.**06 | 2**.**11 | 35**.**65 | 282**.**40 | 11**.**39 |
| Peru | M | 79**.**30 | 5**.**35 | 0**.**06 | 2**.**11 | 58**.**33 | 462**.**00 | 16**.**76 |
| Philippines | L | n/a | 5**.**35 | 0**.**06 | 2**.**11 | 21**.**51 | 285**.**66 | 7**.**94 |
| Saudi Arabia | H | 62**.**37 | 43**.**39 | 1**.**21 | 1**.**06 | 328**.**50 | 840**.**81 | 56**.**08 |
| Senegal | L | n/a | 5**.**35 | 0**.**06 | 2.11 | 8**.**83 | 117**.**22 | 2**.**49 |
| Sierra Leone | L | n/a | 5**.**35 | 0**.**06 | 2**.**11 | 3**.**04 | 40**.**42 | 1**.**90 |
| Singapore | H | 62**.**37 | 43**.**39 | 1**.**21 | 1**.**06 | 746**.**32 | 1,910**.**25 | 144**.**80 |
| Somalia | L | n/a | 5**.**35 | 0**.**06 | 2**.**11 | 3**.**56 | 47**.**25 | 1**.**51 |
| South Africa | M | 79**.**30 | 5**.**35 | 0**.**06 | 2**.**11 | 93**.**47 | 740**.**34 | 15**.**58 |
| South Sudan | L | n/a | 5**.**35 | 0**.**06 | 2**.**11 | 19**.**70 | 261**.**71 | 2**.**00 |
| Sri Lanka | L | n/a | 5**.**35 | 0**.**06 | 2**.**11 | 31**.**06 | 412**.**52 | 10**.**75 |
| Sudan | L | n/a | 5**.**35 | 0**.**06 | 2**.**11 | 90**.**61 | 1,203**.**55 | 10**.**75 |
| Suriname | M | 79**.**30 | 5**.**35 | 0**.**06 | 2**.**11 | 97**.**72 | 774**.**03 | 24**.**60 |
| Tanzania | L | n/a | 5**.**35 | 0**.**06 | 2**.**11 | 7**.**04 | 93**.**45 | 2**.**37 |
| Thailand | M | 79**.**30 | 5**.**35 | 0**.**06 | 2**.**11 | 47**.**71 | 377**.**88 | 15**.**92 |
| Timor-Leste | L | n/a | 5**.**35 | 0**.**06 | 2**.**11 | 5**.**65 | 75**.**03 | 3**.**11 |
| Togo | L | n/a | 5**.**35 | 0**.**06 | 2**.**11 | 5**.**59 | 74**.**22 | 1**.**50 |
| Uganda | L | n/a | 5**.**35 | 0**.**06 | 2**.**11 | 7**.**53 | 99**.**98 | 1**.**85 |
| Venezuela | M | 79**.**30 | 5**.**35 | 0**.**06 | 2**.**11 | 1,866**.**41 | 14,783**.**52 | 33**.**58 |
| Vietnam | L | n/a | 5**.**35 | 0**.**06 | 2**.**11 | 17**.**53 | 232**.**80 | 5**.**78 |
| Yemen | L | n/a | 5**.**35 | 0**.**06 | 2**.**11 | 21**.**86 | 290**.**36 | 3**.**86 |
| Zambia | L | n/a | 5**.**35 | 0**.**06 | 2**.**11 | 16**.**32 | 216**.**77 | 3**.**58 |
| Zimbabwe | L | n/a | 5**.**35 | 0**.**06 | 2**.**11 | 8**.**82 | 117**.**18 | 2**.**44 |

Table S3(c). Country-specific economic parameters - children under 15 years.

|  | **Antibiotic costs** | | | |
| --- | --- | --- | --- | --- |
| **Country** | **Meropenem** | **Ceftazidime** | **Trimethoprim/**  **Sulfamethoxazole** | **Co-Amoxiclav** |
| Angola | n/a | 3**.**44 | 0**.**05 | 1**.**41 |
| Argentina | 59**.**56 | 3**.**44 | 0**.**05 | 1**.**41 |
| Australia | 46**.**78 | 27**.**89 | 0**.**91 | 0**.**71 |
| Bangladesh | n/a | 3**.**44 | 0**.**05 | 1**.**41 |
| Benin | n/a | 3**.**44 | 0**.**05 | 1**.**41 |
| Bhutan | n/a | 3**.**44 | 0**.**05 | 1**.**41 |
| Bolivia | n/a | 3**.**44 | 0**.**05 | 1**.**41 |
| Brazil | 59**.**56 | 3**.**44 | 0**.**05 | 1**.**41 |
| Brunei | 46**.**78 | 27**.**89 | 0**.**91 | 0**.**71 |
| Burkina Faso | n/a | 3**.**44 | 0**.**05 | 1**.**41 |
| Cambodia | n/a | 3**.**44 | 0**.**05 | 1**.**41 |
| Cameroon | n/a | 3**.**44 | 0**.**05 | 1**.**41 |
| Central African Republic | n/a | 3**.**44 | 0**.**05 | 1**.**41 |
| Chad | n/a | 3.44 | 0**.**05 | 1**.**41 |
| China | 59**.**56 | 3**.**44 | 0**.**05 | 1**.**41 |
| Colombia | 59**.**56 | 3**.**44 | 0**.**05 | 1**.**41 |
| Congo, Dem. Rep. | n/a | 3**.**44 | 0**.**05 | 1**.**41 |
| Congo, Rep. | n/a | 3**.**44 | 0**.**05 | 1**.**41 |
| Costa Rica | 59**.**56 | 3**.**44 | 0**.**05 | 1**.**41 |
| Cote d'Ivoire | n/a | 3**.**44 | 0**.**05 | 1**.**41 |
| Cuba | 59**.**56 | 3**.**44 | 0**.**05 | 1**.**41 |
| El Salvador | n/a | 3**.**44 | 0**.**05 | 1**.**41 |
| Equatorial Guinea | 59**.**56 | 3**.**44 | 0.05 | 1**.**41 |
| Eritrea | n/a | 3.44 | 0**.**05 | 1**.**41 |
| Ethiopia | n/a | 3**.**44 | 0**.**05 | 1**.**41 |
| Fiji | 59**.**56 | 3**.**44 | 0**.**05 | 1**.**41 |
| Gabon | 59**.**56 | 3**.**44 | 0.05 | 1**.**41 |
| Gambia | n/a | 3**.**44 | 0**.**05 | 1**.**41 |
| Ghana | n/a | 3**.**44 | 0**.**05 | 1**.**41 |
| Guatemala | n/a | 3**.**44 | 0**.**05 | 1**.**41 |
| Guinea | n/a | 3**.**44 | 0**.**05 | 1**.**41 |
| Guinea-Bissau | n/a | 3**.**44 | 0**.**05 | 1**.**41 |
| Guyana | 59**.**56 | 3**.**44 | 0**.**05 | 1**.**41 |
| Haiti | n/a | 3**.**44 | 0**.**05 | 1**.**41 |
| Honduras | n/a | 3**.**44 | 0**.**05 | 1**.**41 |
| Hong Kong | 46**.**78 | 27**.**89 | 0**.**91 | 0**.**71 |
| India | n/a | 3**.**44 | 0**.**05 | 1**.**41 |
| Indonesia | n/a | 3**.**44 | 0**.**05 | 1**.**41 |
| Iran | 59**.**56 | 3**.**44 | 0**.**05 | 1**.**41 |
| Iraq | 59**.**56 | 3**.**44 | 0**.**05 | 1**.**41 |
| Kenya | n/a | 3**.**44 | 0**.**05 | 1**.**41 |
| Lao | n/a | 3**.**44 | 0**.**05 | 1**.**41 |
| Liberia | n/a | 3**.**44 | 0**.**05 | 1**.**41 |
| Madagascar | n/a | 3**.**44 | 0**.**05 | 1**.**41 |
| Malawi | n/a | 3**.**44 | 0**.**05 | 1**.**41 |
| Malaysia | 59**.**56 | 3**.**44 | 0**.**05 | 1.41 |
| Mali | n/a | 3**.**44 | 0**.**05 | 1**.**41 |
| Mauritania | n/a | 3**.**44 | 0**.**05 | 1**.**41 |
| Mauritius | 59**.**56 | 3**.**44 | 0**.**05 | 1**.**41 |
| Mexico | 59**.**56 | 3.44 | 0**.**05 | 1**.**41 |
| Mozambique | n/a | 3**.**44 | 0**.**05 | 1**.**41 |
| Myanmar | n/a | 3**.**44 | 0**.**05 | 1**.**41 |
| Nepal | n/a | 3**.**44 | 0**.**05 | 1**.**41 |
| Nicaragua | n/a | 3**.**44 | 0**.**05 | 1**.**41 |
| Niger | n/a | 3**.**44 | 0**.**05 | 1**.**41 |
| Nigeria | n/a | 3**.**44 | 0**.**05 | 1**.**41 |
| Oman | 46**.**78 | 27**.**89 | 0**.**91 | 0**.**71 |
| Pakistan | n/a | 3**.**44 | 0**.**05 | 1**.**41 |
| Panama | 59**.**56 | 3**.**44 | 0**.**05 | 1**.**41 |
| Papua New Guinea | n/a | 3**.**44 | 0**.**05 | 1**.**41 |
| Paraguay | 59**.**56 | 3**.**44 | 0**.**05 | 1**.**41 |
| Peru | 59**.**56 | 3**.**44 | 0**.**05 | 1**.**41 |
| Philippines | n/a | 3**.**44 | 0**.**05 | 1**.**41 |
| Saudi Arabia | 46**.**78 | 27**.**89 | 0**.**91 | 0**.**71 |
| Senegal | n/a | 3**.**44 | 0**.**05 | 1**.**41 |
| Sierra Leone | n/a | 3**.**44 | 0**.**05 | 1**.**41 |
| Singapore | 46**.**78 | 27**.**89 | 0**.**91 | 0**.**71 |
| Somalia | n/a | 3**.**44 | 0**.**05 | 1**.**41 |
| South Africa | 59**.**56 | 3**.**44 | 0**.**05 | 1**.**41 |
| South Sudan | n/a | 3**.**44 | 0**.**05 | 1**.**41 |
| Sri Lanka | n/a | 3**.**44 | 0**.**05 | 1**.**41 |
| Sudan | n/a | 3**.**44 | 0**.**05 | 1**.**41 |
| Suriname | 59**.**56 | 3**.**44 | 0**.**05 | 1**.**41 |
| Tanzania | n/a | 3**.**44 | 0**.**05 | 1**.**41 |
| Thailand | 59**.**56 | 3**.**44 | 0**.**05 | 1**.**41 |
| Timor-Leste | n/a | 3**.**44 | 0**.**05 | 1**.**41 |
| Togo | n/a | 3**.**44 | 0**.**05 | 1**.**41 |
| Uganda | n/a | 3**.**44 | 0**.**05 | 1**.**41 |
| Venezuela | 59**.**56 | 3**.**44 | 0**.**05 | 1**.**41 |
| Vietnam | n/a | 3**.**44 | 0**.**05 | 1.41 |
| Yemen | n/a | 3**.**44 | 0**.**05 | 1**.**41 |
| Zambia | n/a | 3**.**44 | 0**.**05 | 1**.**41 |
| Zimbabwe | n/a | 3**.**44 | 0**.**05 | 1**.**41 |

**Table S4. Incremental cost-effectiveness ratio (ICER) for different vaccination strategies (compared to the next most expensive strategy) by region.**

|  |  | |  | |  | |  |  | |  | |  | |  |
| --- | --- | --- | --- | --- | --- | --- | --- | --- | --- | --- | --- | --- | --- | --- |
| **Regions** | | **Willingness to pay by region (Median)** | | **Vac 2**  **(Compared with no vaccine)** | | | | **Vac 3**  **(Compared with Vac 2)** | | | **Vac 4**  **(Compared with Vac 3)** | | | |
|  |  |  |  | **ICER (Mean)** | | **95% Confidence Interval** | | **ICER (Mean)** | **95% Confidence Interval** | | **ICER (Mean)** | | **95% Confidence Interval** | |
| East Asia and Pacific (EAP) | | 4,131 | | 1,036.52 | | (631.62, 1,671.03) | | 4,025.47 | (2,233.04, 6,871.71) | | 5,500.31 | | (3,158.82, 9,217.71) | |
| Latin America & Caribbean (LAC) | | 6,089 | | 5,198.10 | | (2,084.23, 10,737.40) | | 24,983.77 | (10,514.43, 49,055.75) | | 32,869.10 | | (10,514.43, 49,055.75) | |
| Middle East & North Africa (MENA) | | 5,443 | | 83,821.28 | | (25,625.99, 232,599.02) | | 234,322.30 | (66,200.14, 689,749.92) | | 268,994.20 | | (74,641.89, 806,734.23) | |
| Sub-Saharan Africa (SAF) | | 760 | | 288.62 | | (111.91, 576.14) | | 1,397.29 | (611.19, 2,554.87) | | 1,854.62 | | (834.15, 3,392.87) | |
| South Asia (SOA) | | 1,505 | | 365.09 | | (143.32, 796.66) | | 1,807.39 | (750.71, 3,777.04) | | 2,391.33 | | (1,007.70, 4,925.46) | |
|  | |  | |  | |  | |  |  | |  | |  | |
|  |  | |  | |  | |  |  | |  | |  | |  |

**Figure S2. Incremental cost-effectiveness ratio (ICER) for different vaccination strategies (compared to no vaccination) by region.**


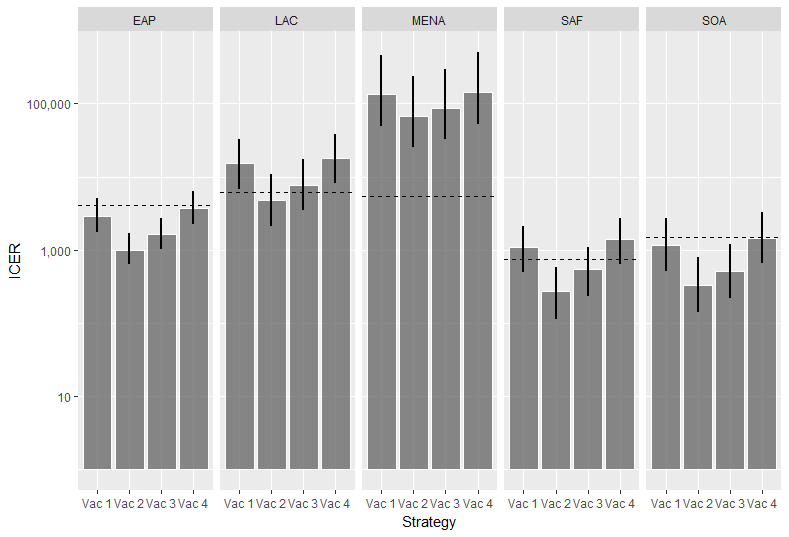


Vaccination strategies; Vac 1: Age >45 with chronic renal disease, Vac 2: Age>45 with diabetes, Vac 3: Age>45 with diabetes, chronic renal disease, or both, and Vac 4: Age>45 (with and without risk factors)

**Table S5. Results of the base case analysis (compared with no vaccination) by country/territory. "Strategy" indicates the optimal cost-effective strategy (Vac 1: Age>45 with chronic renal disease, Vac 2: Age>45 with diabetes, Vac 3: Age>45 with diabetes, chronic renal disease, or both, and Vac 4: Age>45 with or without risk factors).**

|  | Country | Strategy | Cases averted | | Deaths averted | Incremental Costs (USD) | Incremental QALYs | ICER  (USD) | GDP per capita |
| --- | --- | --- | --- | --- | --- | --- | --- | --- | --- |
|  | **East Asia and Pacific (EAP)** | | | |  |  |  |  |  |
|  | Australia | Vac2 | | 4.985 | 1.171 | 438,718 | 20.2 | 21,725 | 56,328 |
|  | Brunei | Vac2 | | 1.189 | 0.369 | 142,613 | 6.0 | 23,785 | 36,608 |
|  | Cambodia | Vac4 | | 172.925 | 95.387 | 1,204,042 | 1,449.2 | 831 | 1,158 |
|  | China | Vac2 | | 227.391 | 82.855 | 4,535,132 | 1,324.7 | 3,423 | 7,925 |
|  | Fiji | Not CE | | - | - | - | - | - | 4,916 |
|  | Hong Kong | Not CE | | - | - | - | - | - | 42,423 |
|  | Indonesia | Vac4 | | 1,584.202 | 808.308 | 13,081,260 | 12,147.1 | 1,077 | 3,346 |
|  | Lao PDR | Vac4 | | 35.748 | 22.130 | 371,108 | 332.3 | 1,117 | 1,812 |
|  | Malaysia | Vac2 | | 76.081 | 22.538 | 1,105,064 | 357.1 | 3,094 | 9,766 |
|  | Myanmar | Vac4 | | 518.332 | 305.921 | 3,456,496 | 4,584.9 | 754 | 1,204 |
|  | Papua New Guinea | Vac3 | | 4.797 | 2.938 | 32,081 | 42.5 | 755 | 2,268 |
|  | Philippines | Vac4 | | 767.589 | 379.753 | 6,650,000 | 5,731.9 | 1,160 | 2,899 |
|  | Singapore | Not CE | | - | - | - | - | - | 52,889 |
|  | Thailand | Vac2 | | 175.131 | 65.639 | 1,390,300 | 1,072.6 | 1,296 | 5,816 |
|  | Timor-Leste | Vac2 | | 0.300 | 0.180 | 1,843 | 2.7 | 676 | 1,134 |
|  | Vietnam | Vac4 | | 829.931 | 374.225 | 6,129,946 | 6,267.9 | 978 | 2,111 |
|  |  |  | | **4,398.600** | **2,161.413** | **38,538,602** | **33,339.2** |  |  |
|  | **South Asia (SOA)** | | |  |  |  |  |  |  |
|  | Bangladesh | Vac3 | | 587.878 | 328.262 | 1,919,514 | 5,236.4 | 367 | 1,212 |
|  | Bhutan | Vac4 | | 1.055 | 0.649 | 10,243 | 10.5 | 974 | 2,532 |
|  | India | Vac2 | | 1,828.998 | 1,094.661 | 5,347,237 | 16,920.6 | 316 | 1,582 |
|  | Nepal | Vac2 | | 15.952 | 8.761 | 36,342 | 133.3 | 273 | 732 |
|  | Pakistan | Vac2 | | 14.142 | 8.319 | 117,138 | 127.6 | 918 | 1,429 |
|  | Sri Lanka | Vac4 | | 150.494 | 49.525 | 1,109,008 | 801.7 | 1,383 | 3,926 |
|  |  |  | | **2,598.518** | **1,490.176** | **8,539,482** | **23,230.2** |  |  |
|  | **Middle East & North Africa (MENA)** | | | |  |  |  |  |  |
|  | Iran | Not CE | | - | - | - | - | - | 5,443 |
|  | Iraq | Not CE | | - | - | - | - | - | 4,629 |
|  | Oman | Not CE | | - | - | - | - | - | 15,645 |
|  | Saudi Arabia | Not CE | | - | - | - | - | - | 20,482 |
|  | Yemen | Vac2 | | 3.864 | 2.303 | 35,655 | 34.3 | 1,040 | 1,408 |
|  |  |  | | **3.864** | **2.303** | **35,655** | **34.3** |  |  |
|  | **Latin America & Caribbean (LAC)** | | | |  |  |  |  |  |
|  | Argentina | Vac2 | | 0.405 | 0.158 | 26,498 | 2.6 | 10,224 | 13,432 |
|  | Bolivia | Vac2 | | 0.369 | 0.199 | 2,674 | 3.3 | 817 | 3,095 |
|  | Brazil | Vac2 | | 33.106 | 12.870 | 1,068,972 | 211.8 | 5,046 | 8,539 |
|  | Colombia | Vac2 | | 4.350 | 1.773 | 130,070 | 29.3 | 4,438 | 6,056 |
|  | Costa Rica | Vac2 | | 0.524 | 0.164 | 18,094 | 2.8 | 6,480 | 10,630 |
|  | Cuba | Not CE | | - | - | - | - | - | 6,790 |
|  | El Salvador | Vac3 | | 4.985 | 1.968 | 43,034 | 32.4 | 1,328 | 4,219 |
|  | Guatemala | Vac3 | | 3.185 | 1.593 | 31,086 | 25.9 | 1,200 | 3,904 |
|  | Guyana | Not CE | | - | - | - | - | - | 4,127 |
|  | Haiti | Not CE | | - | - | - | - | - | 829 |
|  | Honduras | Vac2 | | 2.625 | 1.160 | 10,943 | 19.3 | 567 | 2,496 |
|  | Mexico | Vac2 | | 27.910 | 10.961 | 1,029,558 | 183.3 | 5,616 | 9,009 |
|  | Nicaragua | Vac2 | | 2.607 | 1.203 | 19,591 | 20.0 | 979 | 2,087 |
|  | Panama | Vac2 | | 2.179 | 0.910 | 49,259 | 15.6 | 3,150 | 13,268 |
|  | Paraguay | Not CE | | - | - | - | - | - | 4,161 |
|  | Peru | Vac2 | | 1.096 | 0.449 | 26,116 | 7.4 | 3,534 | 6,122 |
|  | Suriname | Vac2 | | 0.554 | 0.237 | 14,253 | 3.8 | 3,799 | 8,984 |
|  | Venezuela | Vac2 | | 3.895 | 1.513 | 96,571 | 25.0 | 3,870 | 12,265 |
|  |  |  | | **87.791** | **35.159** | **2,566,719** | **582.5** |  |  |
|  | **Sub-Saharan Africa (SAF)** | | |  |  |  |  |  |  |
|  | Angola | Vac2 | | 0.412 | 0.242 | 2,807 | 3.5 | 800 | 4,102 |
|  | Benin | Vac3 | | 15.413 | 9.476 | 54,834 | 138.3 | 396 | 779 |
|  | Burkina Faso | Vac2 | | 9.858 | 6.006 | 22,674 | 87.0 | 261 | 613 |
|  | Cameroon | Vac2 | | 13.752 | 8.327 | 43,170 | 122.7 | 352 | 1,251 |
|  | Central African Republic | Not CE | | - | - | - | - | - | 307 |
|  | Chad | Vac2 | | 6.916 | 4.226 | 18,066 | 61.5 | 294 | 776 |
|  | Congo, Dem. Rep. | Not CE | | - | - | - | - | - | 456 |
|  | Congo, Rep. | Vac4 | | 21.569 | 13.337 | 171,832 | 203.5 | 844 | 1,851 |
|  | Cote d'Ivoire | Vac3 | | 31.404 | 19.325 | 117,965 | 272.3 | 433 | 1,399 |
|  | Equatorial Guinea | Vac2 | | 0.115 | 0.077 | 3,624 | 1.1 | 3,159 | 11,121 |
|  | Eritrea | Not CE | | - | - | - | - | - | 544 |
|  | Ethiopia | Vac2 | | 4.452 | 2.644 | 21,941 | 40.4 | 543 | 619 |
|  | Gabon | Vac2 | | 3.969 | 2.470 | 268,112 | 37.9 | 7,078 | 8,312 |
|  | Gambia | Not CE | | - | - | - | - | - | 441 |
|  | Ghana | Vac2 | | 6.089 | 3.710 | 20,060 | 54.5 | 368 | 1,381 |
|  | Guinea | Vac2 | | 27.848 | 17.090 | 40,964 | 255.2 | 161 | 531 |
|  | Guinea-Bissau | Vac2 | | 1.417 | 0.864 | 3,359 | 13.0 | 259 | 573 |
|  | Kenya | Vac2 | | 2.317 | 1.390 | 10,766 | 20.3 | 531 | 1,377 |
|  | Liberia | Vac2 | | 6.935 | 4.302 | 10,551 | 64.3 | 164 | 456 |
|  | Madagascar | Vac2 | | 20.979 | 15.067 | 43,317 | 227.3 | 191 | 412 |
|  | Malawi | Not CE | | - | - | - | - | - | 381 |
|  | Mali | Vac2 | | 5.326 | 3.269 | 12,297 | 47.9 | 256 | 744 |
|  | Mauritania | Vac2 | | 0.492 | 0.298 | 2,980 | 4.4 | 671 | 1,371 |
|  | Mauritius | Not CE | | - | - | - | - | - | 9,117 |
|  | Mozambique | Vac2 | | 3.491 | 2.083 | 14,593 | 30.9 | 473 | 525 |
|  | Niger | Not CE | | - | - | - | - | - | 359 |
|  | Nigeria | Vac4 | | 1,107.949 | 684.116 | 9,217,842 | 9,612.5 | 959 | 2,640 |
|  | Senegal | Vac2 | | 0.998 | 0.599 | 4,757 | 9.0 | 531 | 911 |
|  | Sierra Leone | Vac2 | | 8.563 | 5.295 | 13,261 | 85.0 | 156 | 693 |
|  | Somalia | Vac2 | | 1.298 | 0.786 | 5,886 | 11.6 | 506 | 552 |
|  | South Africa | Vac2 | | 0.755 | 0.465 | 33,846 | 6.9 | 4,930 | 5,692 |
|  | South Sudan | Not CE | | - | - | - | - | - | 731 |
|  | Sudan | Vac2 | | 2.187 | 1.270 | 18,565 | 19.4 | 955 | 2,089 |
|  | Tanzania | Vac2 | | 4.266 | 2.543 | 13,228 | 36.7 | 361 | 865 |
|  | Togo | Not CE | | - | - | - | - | - | 548 |
|  | Uganda | Not CE | | - | - | - | - | - | 676 |
|  | Zambia | Vac2 | | 2.672 | 1.598 | 11,075 | 24.2 | 458 | 1,308 |
|  | Zimbabwe | Not CE | | - | - | - | - | - | 890 |
|  |  |  | | **1,311.441** | **810.873** | **10,202,372** | **11,491.3** |  |  |
|  | Total from all regions | | | **8,400.214** | **4,499.923** | **59,882,830** | **68,677.5** |  |  |

**Figure S3. Results from a sensitivity analysis assuming 50% vaccine protective efficacy.**

Figure S3(a). Optimal cost-effective vaccination strategy by geography with 50% protective efficacy.


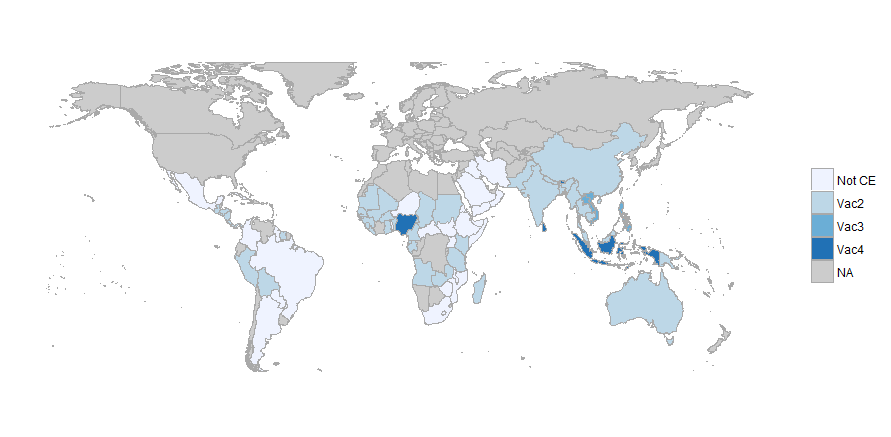


Figure S3(b). Incremental cost-effectiveness ratio for each vaccine strategy by region with 50% protective efficacy.


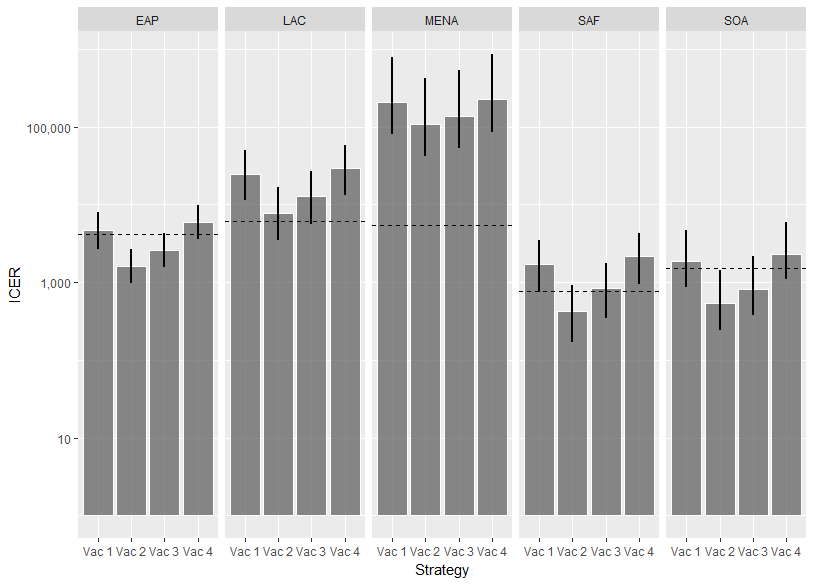


Figure S3(c). Map showing number of lives saved by geography per 100,000 diabetic >45 years vaccinated in environmentally suitable regions (strategy Vac 2) with 50% vaccine efficacy.


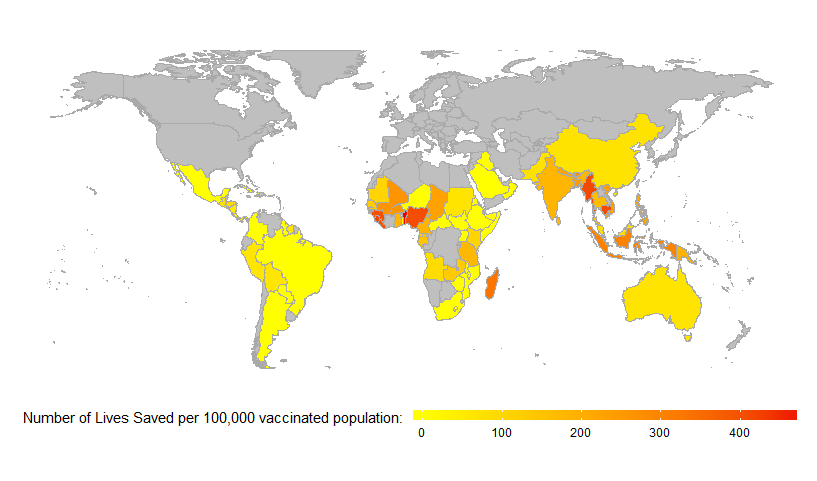


Figure S3(d). Bar plot showing total number of death averted by region for each vaccination strategy with 50% protective efficacy.


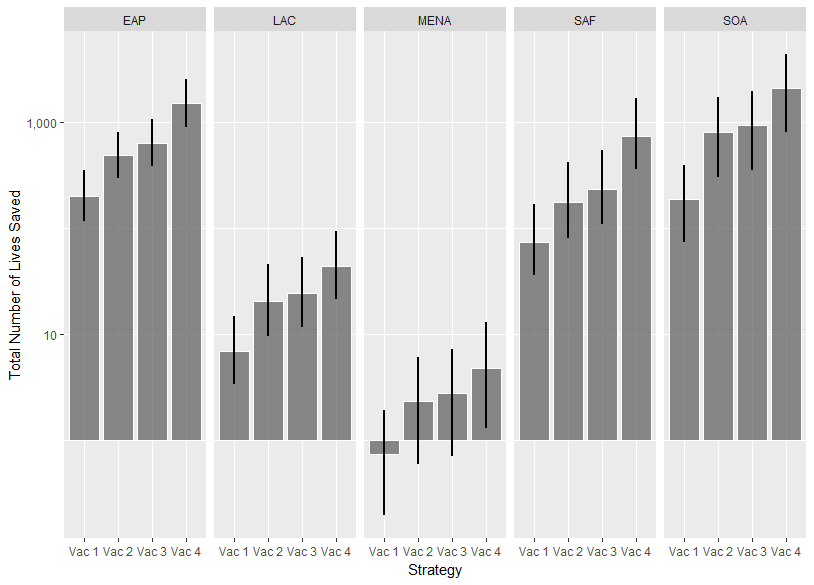


Figure S3(e). Bar plot showing net costs of vaccination by region for each vaccination strategy with 50% protective efficacy.


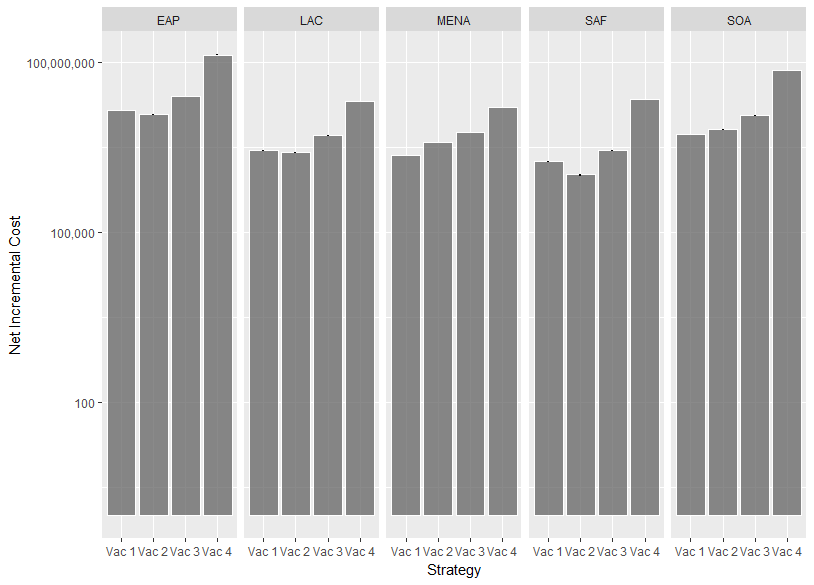


Figure S3(f). Incremental cost-effectiveness ratio (ICER) of vaccinating the population over 45 years with diabetes (strategy Vac 2 with 50% protective efficacy), as a proportion of each geography’s GDP per capita.


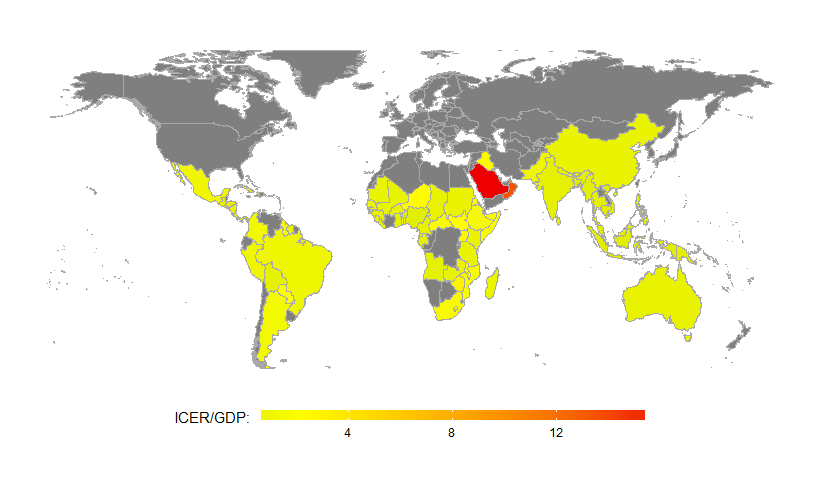


Figure S3(g). ICER by region of each strategy compared to the next best strategy (Vac 2 compared with no vaccination, Vac 3 compared with Vac 2, and Vac 4 compared with Vac 3)


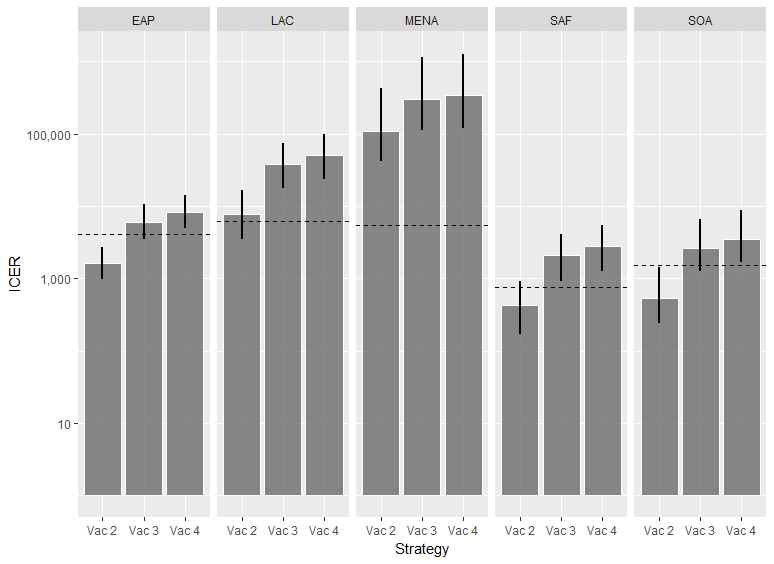


**Table S6. Results** **of all countries/territories from the sensitivity analysis, 50% vaccine protective efficacy. Vaccination strategies; Vac 1: Age>45 with chronic renal disease, Vac 2: Age>45 with diabetes, Vac 3: Age>45 with either chronic renal disease or diabetes, and Vac 4: Age>45 (with and without risk factors)**

| **Country** | **Cost-effective strategy** | **Case averted** | **Death averted** | **Incremental Costs (USD)** | **Incremental QALYs** | **ICER**  **(USD/ QALY gained)** | **WTP**  **(GDP per capita)** |
| --- | --- | --- | --- | --- | --- | --- | --- |
|  |  |  |  |  |  |  |  |
|  | | |  |  |  |  |  |
| **East Asia and Pacific (EAP)** | | |  |  |  |  |  |
| Australia | Vac2 | 3.124 | 0.73 | 443,328 | 12.7 | 35,030.7 | 56,328 |
| Brunei | Not CE | - | - | - | - | - | 36,608 |
| Cambodia | Vac2 | 19.599 | 10.81 | 37,925 | 164.3 | 230.9 | 1,158 |
| China | Vac2 | 144.471 | 52.64 | 4,558,626 | 841.6 | 5,416.5 | 7,925 |
| Fiji | Not CE | - | - | - | - | - | 4,916 |
| Hong Kong | Not CE | - | - | - | - | - | 42,423 |
| Indonesia | Vac4 | 1,055.082 | 538.33 | 13,166,405 | 8,089.9 | 1,627.5 | 3,346 |
| Lao PDR | Vac3 | 8.220 | 5.09 | 52,433 | 76.4 | 686.2 | 1,812 |
| Malaysia | Vac2 | 47.283 | 14.01 | 1,121,794 | 222.0 | 5,054.2 | 9,766 |
| Myanmar | Vac2 | 92.131 | 54.38 | 218,452 | 814.9 | 268.1 | 1,204 |
| Papua New Guinea | Vac2 | 2.268 | 1.39 | 12,215 | 20.1 | 608.3 | 2,268 |
| Philippines | Vac3 | 254.037 | 125.68 | 1,487,323 | 1,897.1 | 784.0 | 2,899 |
| Singapore | Not CE | - | - | - | - | - | 52,889 |
| Thailand | Vac2 | 106.439 | 39.89 | 1,411,153 | 651.9 | 2,164.8 | 5,816 |
| Timor-Leste | Vac2 | 0.184 | 0.11 | 1,848 | 1.7 | 1,106.7 | 1,134 |
| Vietnam | Vac3 | 199.272 | 89.85 | 905,022 | 1,505.0 | 601.4 | 2,111 |
|  |  | **1,932.109** | **932.92** | **23,416,524** | **14,297.4** |  |  |
|  | |  |  |  |  |  |  |
| **South Asia (SOA)** | |  |  |  |  |  |  |
| Bangladesh | Vac2 | 312.047 | 174.242 | 1,015,131 | 2,779.499 | 365.2 | 1,212 |
| Bhutan | Vac4 | 0.674 | 0.415 | 10,294 | 6.719 | 1,532.1 | 2,532 |
| India | Vac2 | 1,108.080 | 663.189 | 5,411,769 | 10,251.465 | 527.9 | 1,582 |
| Nepal | Vac2 | 9.937 | 5.458 | 36,579 | 83.054 | 440.4 | 732 |
| Pakistan | Vac2 | 9.401 | 5.530 | 117,528 | 84.848 | 1,385.1 | 1,429 |
| Sri Lanka | Vac4 | 97.290 | 32.016 | 1,116,956 | 518.342 | 2,154.9 | 3,926 |
|  |  | **1,537.430** | **880.851** | **7,708,257** | **13,723.9** |  |  |
|  | | |  |  |  |  |  |
| **Middle East & North Africa (MENA)** | | |  |  |  |  |  |
| Iran | Not CE | - | - | - | - | - | 5,443 |
| Iraq | Not CE | - | - | - | - | - | 4,629 |
| Oman | Not CE | - | - | - | - | - | 15,645 |
| Saudi Arabia | Not CE | - | - | - | - | - | 20,482 |
| Yemen | Not CE | - | - | - | - | - | 1,408 |
|  |  | - | - | - | - |  |  |
|  | | |  |  |  |  |  |
| **Latin America & Caribbean (LAC)** | | |  |  |  |  |  |
| Argentina | Not CE | - | - | - | - | - | 13,432 |
| Bolivia | Vac2 | 0.227 | 0.122 | 2,692 | 2.0 | 1,339.3 | 3,095 |
| Brazil | Not CE | - | - | - | - | - | 8,539 |
| Colombia | Not CE | - | - | - | - | - | 6,056 |
| Costa Rica | Vac2 | 0.342 | 0.107 | 18,193 | 1.8 | 9,982.5 | 10,630 |
| Cuba | Not CE | - | - | - | - | - | 6,790 |
| El Salvador | Vac2 | 2.681 | 1.058 | 20,505 | 17.4 | 1,176.6 | 4,219 |
| Guatemala | Vac2 | 1.586 | 0.793 | 14,884 | 12.9 | 1,154.0 | 3,903 |
| Guyana | Not CE | - | - | - | - | - | 4,127 |
| Haiti | Not CE | - | - | - | - | - | 829 |
| Honduras | Vac2 | 1.639 | 0.724 | 11,060 | 12.0 | 918.3 | 2,496 |
| Mexico | Not CE | - | - | - | - | - | 9,009 |
| Nicaragua | Vac2 | 1.697 | 0.783 | 19,669 | 13.0 | 1,509.6 | 2,087 |
| Panama | Vac2 | 1.368 | 0.572 | 49,685 | 9.8 | 5,061.1 | 13,268 |
| Paraguay | Not CE | - | - | - | - | - | 4,161 |
| Peru | Vac2 | 0.688 | 0.282 | 26,256 | 4.6 | 5,653.4 | 6,122 |
| Suriname | Vac2 | 0.360 | 0.154 | 14,356 | 2.4 | 5,882.8 | 8,984 |
| Venezuela, RB | Vac2 | 2.413 | 0.937 | 107,089 | 15.5 | 6,927.0 | 12,265 |
|  |  | **13.002** | **5.533** | **284,389** | **91.6** |  |  |
|  | |  |  |  |  |  |  |
| **Sub-Saharan Africa (SAF)** | |  |  |  |  |  |  |
| Angola | Vac2 | 0.259 | 0.152 | 2,873 | 2.2 | 1,304.5 | 4,102 |
| Benin | Vac2 | 5.397 | 3.318 | 10,426 | 48.4 | 215.3 | 779 |
| Burkina Faso | Vac2 | 6.066 | 3.696 | 22,817 | 53.6 | 426.0 | 613 |
| Cameroon | Vac2 | 8.736 | 5.290 | 43,510 | 78.0 | 558.1 | 1,251 |
| Central African Republic | Not CE | - | - | - | - | - | 307 |
| Chad | Vac2 | 4.286 | 2.619 | 18,195 | 38.1 | 477.3 | 776 |
| Congo, Dem. Rep. | Not CE | - | - | - | - | - | 456 |
| Congo, Rep. | Vac4 | 13.834 | 8.554 | 173,126 | 130.5 | 1,326.4 | 1,851 |
| Cote d'Ivoire | Vac2 | 14.477 | 8.909 | 48,372 | 125.5 | 385.4 | 1,399 |
| Equatorial Guinea | Vac2 | 0.071 | 0.047 | 3,722 | 0.7 | 5,297.6 | 11,121 |
| Eritrea | Not CE | - | - | - | - | - | 544 |
| Ethiopia | Not CE | - | - | - | - | - | 619 |
| Gabon | Vac2 | 1.105 | 0.687 | 28,971 | 10.5 | 2,747.7 | 8,311 |
| Gambia, The | Not CE | - | - | - | - | - | 441 |
| Ghana | Vac2 | 3.813 | 2.323 | 20,246 | 34.1 | 593.2 | 1,381 |
| Guinea | Vac2 | 17.443 | 10.705 | 41,431 | 159.8 | 259.2 | 531 |
| Guinea-Bissau | Vac2 | 0.945 | 0.576 | 3,371 | 8.6 | 389.9 | 573 |
| Kenya | Vac2 | 1.362 | 0.817 | 10,828 | 11.9 | 907.6 | 1,377 |
| Liberia | Vac2 | 4.428 | 2.746 | 10,614 | 41.0 | 258.6 | 456 |
| Madagascar | Vac2 | 13.188 | 9.471 | 43,625 | 142.9 | 305.4 | 412 |
| Malawi | Not CE | - | - | - | - | - | 381 |
| Mali | Vac2 | 3.216 | 1.974 | 12,382 | 29.0 | 427.6 | 744 |
| Mauritania | Vac2 | 0.303 | 0.184 | 2,993 | 2.7 | 1,092.9 | 1,371 |
| Mauritius | Not CE | - | - | - | - | - | 9,117 |
| Mozambique | Not CE | - | - | - | - | - | 525 |
| Niger | Not CE | - | - | - | - | - | 359 |
| Nigeria | Vac4 | 718.258 | 443.497 | 9,268,098 | 6,231.8 | 1,487.2 | 2,640 |
| Senegal | Vac2 | 0.628 | 0.377 | 4,777 | 5.6 | 847.5 | 911 |
| Sierra Leone | Vac2 | 5.256 | 3.250 | 13,362 | 52.2 | 256.0 | 693 |
| Somalia | Not CE | - | - | - | - | - | 552 |
| South Africa | Not CE | - | - | - | - | - | 5,692 |
| South Sudan | Not CE | - | - | - | - | - | 731 |
| Sudan | Vac2 | 1.392 | 0.809 | 18,887 | 12.4 | 1,526.1 | 2,089 |
| Tanzania | Vac2 | 2.661 | 1.586 | 13,301 | 22.9 | 581.7 | 865 |
| Togo | Not CE | - | - | - | - | - | 548 |
| Uganda | Not CE | - | - | - | - | - | 676 |
| Zambia | Vac2 | 1.579 | 0.944 | 11,169 | 14.3 | 781.2 | 1,308 |
| Zimbabwe | Not CE | - | - | - | - | - | 890 |
|  |  | **828.704** | **512.533** | **9,827,095** | **7,256.9** |  |  |
| **Total from all regions** | | **4,311.246** | **2,331.836** | **41,236,266** | **35,369.8** |  |  |
